# Recapitulating dynamic ECM ligand presentation at biomaterial interfaces: Molecular strategies and biomedical prospects

**DOI:** 10.1002/EXP.20210093

**Published:** 2022-01-24

**Authors:** Wenbo He, Qinghe Wang, Xiaohua Tian, Guoqing Pan

**Affiliations:** ^1^ Institute for Advanced Materials School of Materials Science and Engineering Jiangsu University Zhenjiang P. R. China; ^2^ School of Chemistry and Chemical Engineering Jiangsu University Zhenjiang P. R. China

**Keywords:** cell behavior regulation, dynamic chemistries, reversible ligand presentation

## Abstract

The extracellular matrix (ECM) provides not only physical support for the tissue structural integrity, but also dynamic biochemical cues capable of regulating diverse cell behaviors and functions. Biomaterial surfaces with dynamic ligand presentation are capable of mimicking the dynamic biochemical cues of ECM, showing ECM‐like functions to modulate cell behaviors. This review paper described an overview of present dynamic biomaterial interfaces by focusing on currently developed molecular strategies for dynamic ligand presentation. The paradigmatic examples for each strategy were separately discussed. In addition, the regulation of some typical cell behaviors on these dynamic biointerfaces including cell adhesion, macrophage polarization, and stem cell differentiation, and their potential applications in pathogenic cell isolation, single cell analysis, and tissue engineering are highlighted. We hope it would not only clarify a clear background of this field, but also inspire to exploit novel molecular strategies and more applications to match the increasing demand of manipulating complex cellular processes in biomedicine.

## Graphical abstract

Mimicking the dynamic ligand presentation in extracellular matrix is crucial to advanced biomaterial design. This review summarizes currently developed dynamic biomaterial interfaces with reversible display of ligands through different molecular strategies. The paradigmatic examples for each strategy as well as their applications in cell regulation are highlighted.

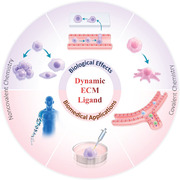

## 1 INTRODUCTION

**FIGURE 1:**
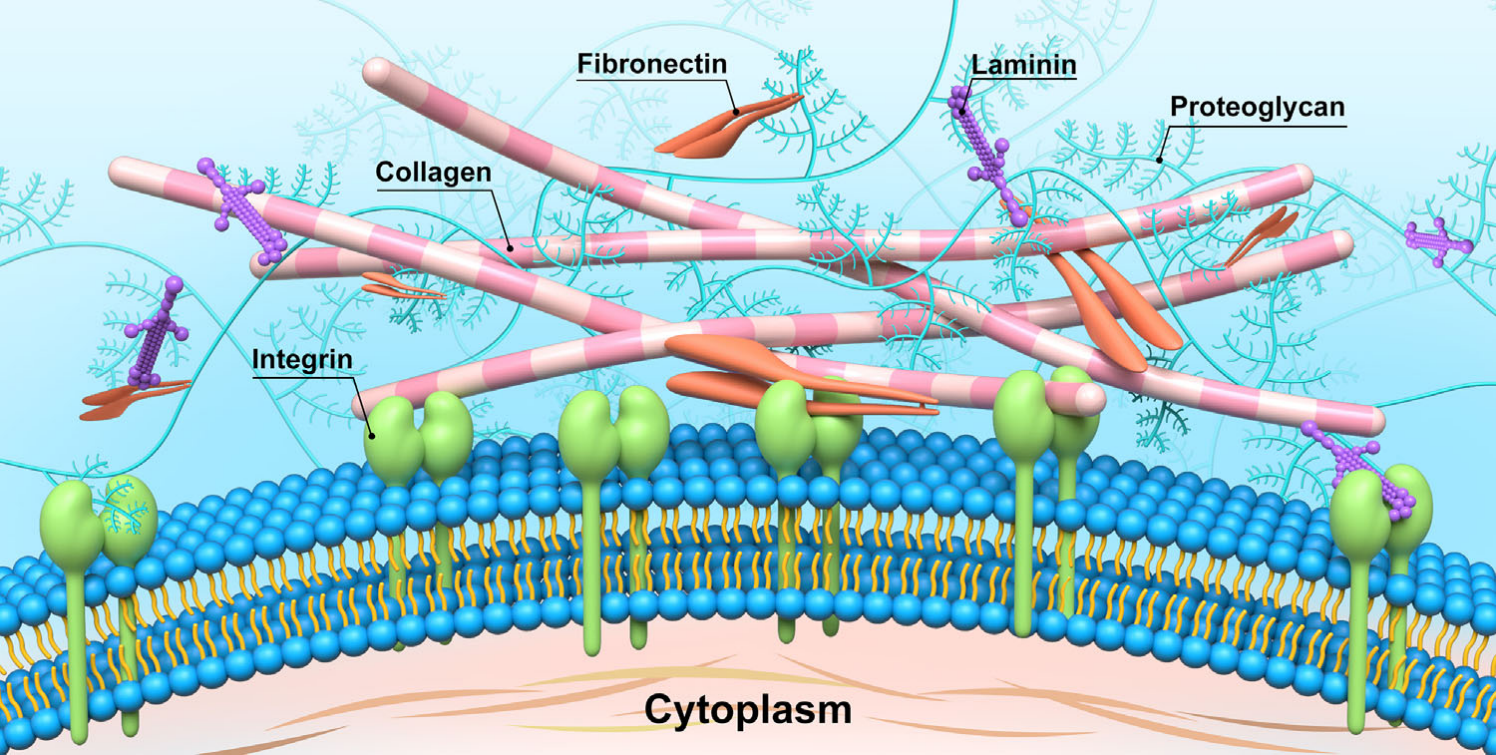
The major components of natural ECM and typical biochemical cues to dynamically interact with and recognize the cell membrane receptors

The extracellular matrix (ECM) possesses highly complex fibrous network, which is mainly composed of plenty of proteins, glycosaminoglycans, proteoglycans, and requested growth factors (Figure 1).^[^ ^1^ ^]^ The ECM is present within all tissues and organs. It provides not only static scaffolds contributing to the mechanical stability of tissues and organs but also dynamic biophysical and biochemical cues for directing a diverse set of cellular functions. Among these dynamics, reversible bioligand presentation represents the most typical dynamic biochemical cues in the ECM. Reversible interactions between the ECM bioligands and cell membrane receptors exist in various cellular processes that are closely related to physiological activities, pathological changes, and aging processes.^[^ ^2^ ^]^ Specific cell signaling and intracellular cascades will be induced as a result of the ECM remodeling processes, which are capable of controlling almost all kind of cell behaviors (e.g., adhesion, migration, polarity, proliferation, differentiation, and apoptosis).^[^ ^3^ ^]^ Clearly, the physiological and pathological processes involve typical dynamic bioligand presentation in the ECM and play an important role in tissue homeostasis, growth, and wound healing.^[^ ^4^ ^]^

As artificial substitutions of the ECM, synthetic biomaterials have been extensively developed for mimicking the ECM functions of cell settlement, organization, and differentiation. These synthetic ECM substitutions, known as biomaterials, have shown promise in fundamental biology, regenerative medicine, medical diagnosis, and so on. The past few decades have witnessed a great advance in the development of biomaterials, while the exploration of advanced biomaterials with ECM‐like functions, especially with the dynamic biochemical cues (i.e., dynamic bioligand presentation) is a field that is still in its infancy. Previous studies revealed that the static physical properties of a biomaterial like the stiffness, topography, and bioligand density can affect various cellular behaviors. In fact, the dynamic biochemical cues involved in a biomaterial will provide a more direct stimulation in cell responses. These ECM‐like dynamic biomaterials are capable of reproducing the reversible bioactivity of natural ECM, thus showing distinct advantages to modulate cell–biomaterial interactions and adapt the development of cellular processes as compared with traditional static biomaterials.

To mimic the biochemical dynamics of ECM in biomaterials, dynamic molecular strategies, including the noncovalent bonds and dynamic covalent bonds, have been constantly exploited for dynamic bioligand presentation.^[^ ^5^ ^]^ Current studies on this topic are mainly based on dynamic biomolecular modification on the biomaterial interfaces.^[^ ^6^ ^]^ These sophisticated dynamic biointerfaces with reversible surface bioactivity can modulate cell–material interactions in a controlled manner, thus showing great significance in cell‐biology and biomedicine engineering.^[^ ^4^, ^7^ ^]^ For example, biomaterial surfaces with dynamic bioligands can manipulate cell behaviors by changing external conditions, which allows for the exploration of in vitro and even in vivo cell biology with real‐time monitoring.^[^ ^4^, ^8^ ^]^ Biomaterials with dynamic surface bioactivities are the most ideal cell growth support materials in tissue engineering and regenerative medicine, the surface engineered dynamic biochemical signals favors controlling desirable cell activities and even facilitating the morphogenesis in tissues and organs.^[^ ^9^ ^]^ In addition, the dynamic biomaterial surfaces can also be used to capture and release specific biomolecules or cells, which also show great promise in molecular and cell‐based medical diagnosis.^[^ ^10^ ^]^

**FIGURE 2:**
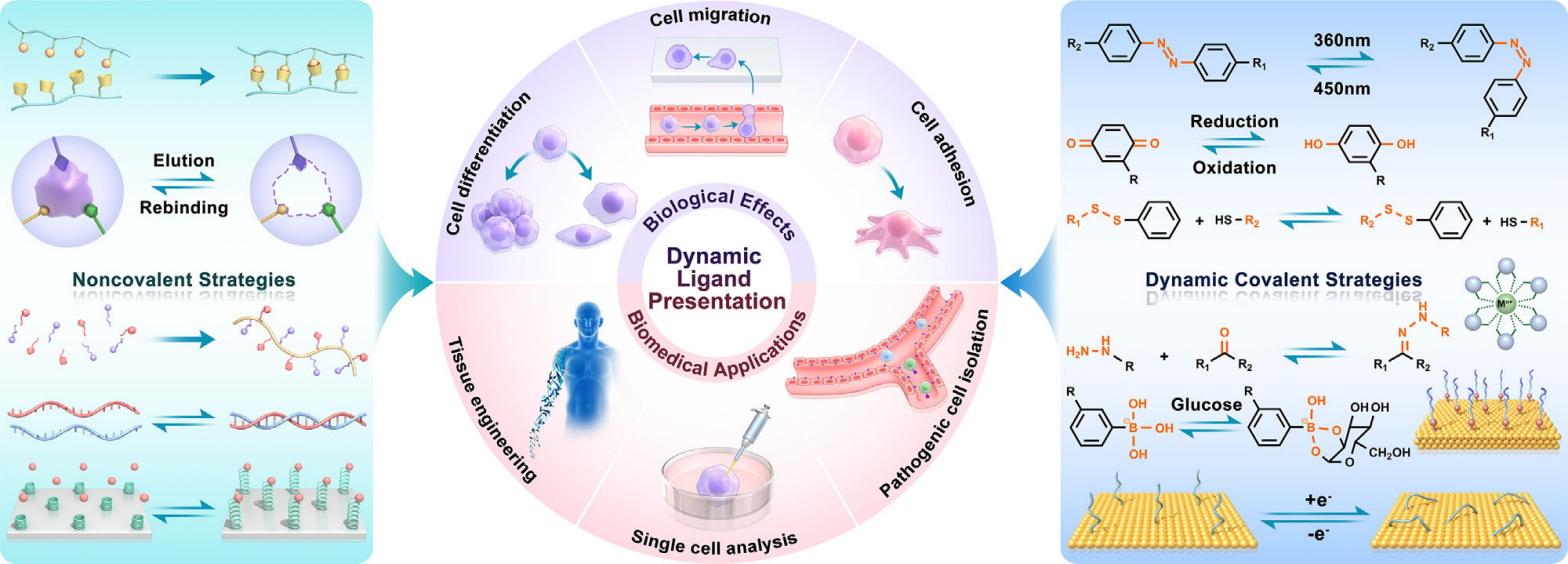
General overview of current dynamic strategies for mimicking the bioligand presentation of natural ECM and their biomedical prospects from the biomaterial point of view

Here, an overview of dynamic biointerfaces was presented by focusing on the reversible molecular strategies and their biological effects/applications (Figure 2). We first elucidated on currently developed strategies for reversible biomolecular modification and dynamic biointerface fabrication. The noncovalent and dynamic covalent chemistries involved in these dynamic biointerfaces were discussed separately. Then, we shifted the attention to the biomedical potentials of dynamic biointerfaces. Several typical cell behaviors including cell adhesion, macrophage polarization and stem cell differentiation, and potential applications in pathogenic cell isolation, single cell analysis, and tissue engineering using these dynamic biointerfaces were highlighted.^[^ ^11^ ^]^ Finally, we gave a forecast in this subject based on existing issues and future developments in biomedical applications. We expect this review paper would not only offer a clear background of dynamic biointerfaces for the relevant researchers, but also with inspirations to develop new molecular strategies for mimicking the dynamic ECM bioligand and for matching the increasing demand to manipulate complex cellular processes in biomedicine.

## 2 DYNAMIC MOLECULAR STRATEGIES FOR BIOLIGAND PRESENTATION

To date, reversible molecular strategies have been proven to be able to introduce bioactive molecules on the biomaterial surfaces and to follow the example of dynamic bioligands in natural ECM.^[^ ^7e,g^ ^,^ ^12^ ^]^ Bioactive ligands including antibodies,^[^ ^13^ ^]^ peptides,^[^ ^14^ ^]^ aptamers,^[^ ^15^ ^]^ and some cell‐binding small molecules^[^ ^16^ ^]^ have been dynamically linked on the biomaterial surfaces via reversible molecular interactions. External stimuli (e.g., light,^[^ ^13^, ^17^ ^]^ temperature,^[^ ^18^ ^]^ pH,^[^ ^19^ ^]^ electrical,^[^ ^20^ ^]^ enzyme,^[^ ^13^, ^15^, ^21^ ^]^ and so on) can specifically trigger the breakage or structural change of the reversible molecular linkers, thus leading to a reverse of the bioligand accessibility and subsequently dynamic transition of the cell behaviors. As mentioned above, reversible molecular strategies could be classified into two major categories, the dynamic covalent interactions and the congenitally reversible noncovalent interactions, according to the types of chemical bonds. Considering the biocompatibility or bioapplicability, currently available covalent methods for dynamic regulation of cell behaviors are mainly based on several typical dynamic covalent chemistries, including photoisomerization, photocleavage, electric transformation, thiol–disulfide exchanging, phenylboronate esters, acid‐cleavable hydrazone, enzymolysis, and metal coordination.^[^ ^7^, ^22^ ^]^ In contrast, the noncovalent methods are relatively milder.^[^ ^23^ ^]^ The host–guest supramolecular interactions, DNA base pairing, tunable intermolecular interactions as well as the molecular imprinting and phage display‐derived biomolecular recognition all have been successfully used to modulate reversible cell–material interactions. Next, we will discuss these reversible molecular strategies for dynamic bioapplicability bioligand presentation.

### 2.1 Dynamic covalent strategies

#### 2.1.1 Azobenzene photoisomerization

Photoisomeric molecules (such as azobenzene,^[^ ^16^, ^24^ ^]^ stilbenes,^[^ ^25^ ^]^ and spiropyrans^[^ ^26^ ^]^) undergo a reversible molecular isomerization in at least two structural states under different light radiations, showing great potentials in optical switching systems and molecular electronics.^[^ ^22^, ^27^ ^]^ Azobenzene photoisomerization represents one of the most widely used dynamic chemistry for the construction of photoresponsive dynamic molecules or materials. In the azobenzene photoisomerization, no chemical bonds are broken, but the molecule structure changes under different UV light exposure (e.g., *cis–trans*). Such a kind of azobenzene isomerization shows the possibility to change intermolecular interactions.

**FIGURE 3:**
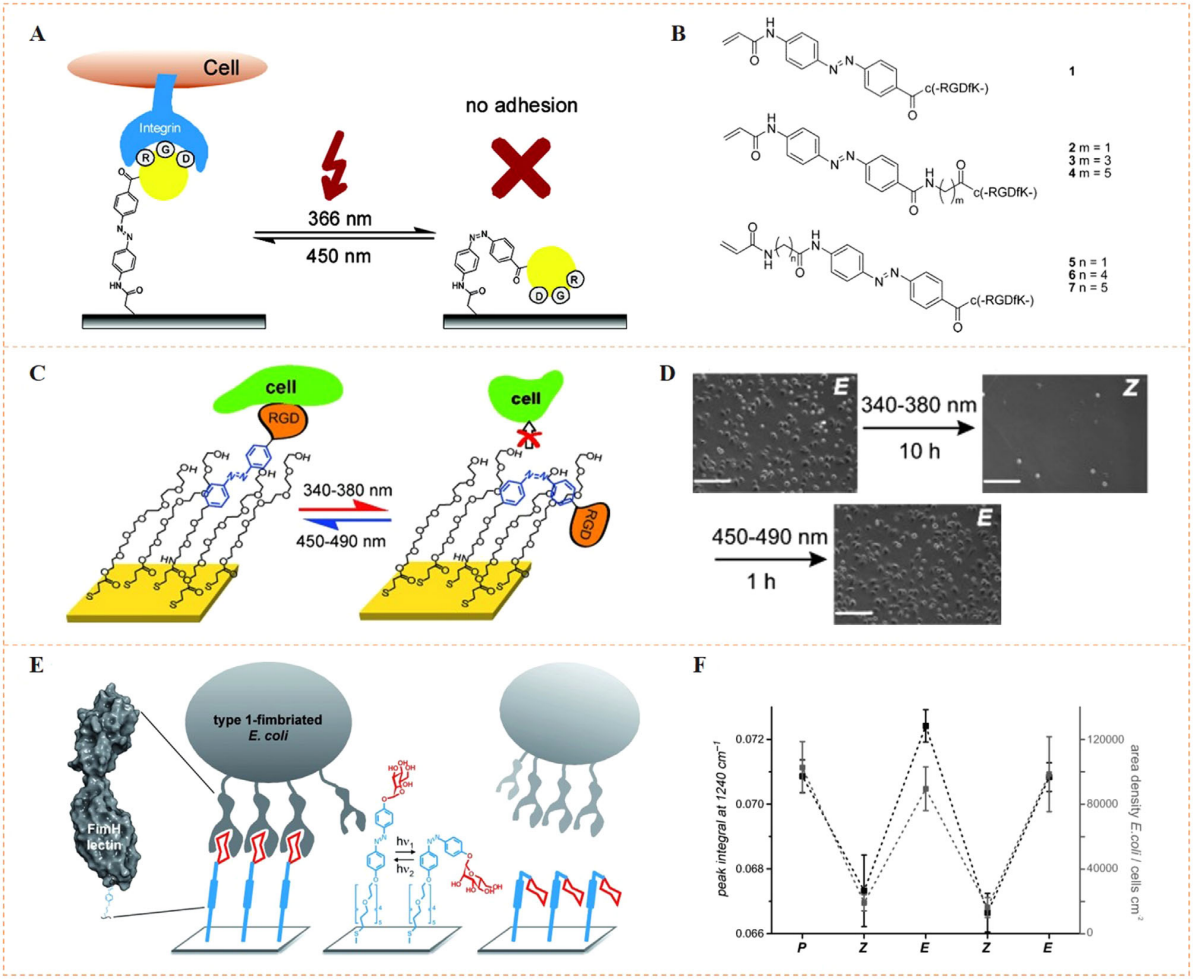
Dynamic presentation of bioligands through azobenzene‐based photoisomerization. (A) The schematic diagram of an azobenzene‐based photoswitchable substrate. (B) A set of cyclic RGD peptide‐capped photoswitchable molecules used in this work. Reproduced with permission.^[^ ^28^ ^]^ Copyright 2005, American Chemical Society. (C) Schematic illustration of azobenzene‐linked SAM for the photo‐reversible control of cell adhesion. (D) UV‐triggered cell attachment and detachment at the biointerface. Reproduced with permission.^[^ ^17c^ ^]^ Copyright 2009, John Wiley and Sons. (E) Schematic diagram of dynamic oriented α‐D‐mannoside surface for photocontrol adhesion of type 1‐fimbriated *Escherichia coli*. (F) Reversible bacterial adhesion to the surface through cis‐trans isomerization cycles. The infrared band at 1240 cm^−1^ is indicative for the aryl O(‐mannosyl) vibration is represented by the infrared band at 1240 cm^−1^. Reproduced with permission.^[^ ^29^ ^]^ Copyright 2014, John Wiley and Sons

The earlier idea to use azobenzene‐functionalized molecule to control bioligand display was reported by Jörg and co‐workers (Figure 3A).^[^ ^28^ ^]^ In their work, a set of acrylamide‐modified RGD (Arg‐Gly‐Asp) cyclopeptide containing the photoswitchable azobenzene with different length as the spacers between the acrylamide group and the peptide were synthesized. Since the RGD sequence is a fibronectin‐derived, integrin‐targeting molecule that mediates specific cell adhesion, the synthetic peptides were then coated on a poly(methyl methacrylate) surface to investigate the photocontrolled cell adhesion. The author found that the photo‐triggered *cis* to *trans* isomerization of azobenzene could regulate the distance between RGD‐containing peptides and the surface, which resulted in different levels of inaccessibility of the cell‐binding RGD motifs for cell binding and subsequently different changes in cell adhesion. To improve the control precision and reversibility of photoisomerization for cell regulation, Liu et al. prepared a self‐assembled monolayer (SAM) using azobenzene‐linked RGD motifs for the photo‐reversible control of cell adhesion (Figure 3B).^[^ ^17c^ ^]^ Different from the structural randomness of azobenzene‐containing polymer surfaces,^[^ ^22^, ^28^ ^]^ the reported SAM could generate molecularly well‐defined surfaces, which would facilitate reversible presentation of RGD motifs with improved precision and enhanced efficiency. The results confirmed that the *cis* and *trans* states on the SAM surface were highly reversible under different UV light, thus allowing reversible control of cell adhesion. Recently, a similar photoresponsive SAM with dynamically oriented α‐d‐mannoside was reported for photocontrol of bacteria adhesion (Figure 3C).^[^ ^29^ ^]^ α‐d‐mannoside is an *Escherichia coli*‐targeting molecule, which can be recognized by type 1 fimbriae of bacteria. The photoswitchable glyco‐SAM showed strong adhesion under light of 450 nm, while the 365 nm light irradiation resulted in a dramatically decreased number in the adherent bacteria.

The above work suggests that photoisomerization is an efficient molecular strategy to reversibly present bioligands and regulate cell behaviors. Moreover, it also provides a hint that such kind of photocontrolled technique may be used to dynamically and spatially modulate the behaviors of cell patterns or single cell. For example, Laith et al. designed an azobenzene‐mediated photoresponsive biointerface for control of single cell adhesion.^[^ ^24c^ ^]^ Besides cell adhesion, cell migration is also an important behavior that is associated with biology and pathology (e.g., tissue repair and cancer metastasis). Recently, Chiara et al. reported an azobenzene‐based surface with sinusoidal topography capable of guiding collective migration of epithelial cells.^[^ ^30^ ^]^ The work implied that using azobenzene‐mediated bioligand presentation may also allow spatial display surface biochemical cues to control the mobility of cells, leading to photocontrolled cell migration. We believe that these potential uses will be successively achieved in a near future.

#### 2.1.2 Nitrophenyl photocleavage

**FIGURE 4:**
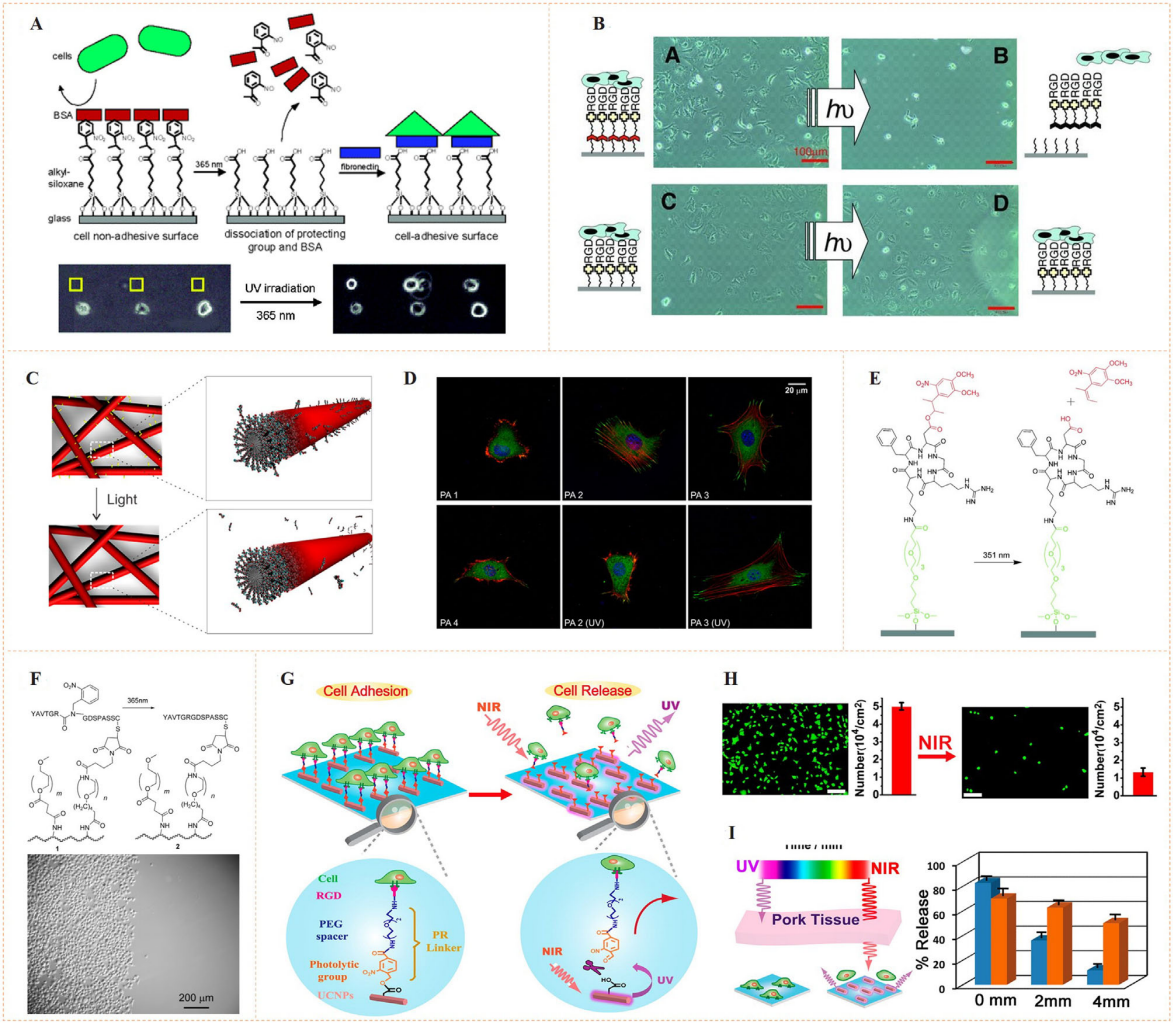
Dynamic presentation of bioligands through nitrophenyl photocleavage. (A) Schematic representation of photoactivation of cell adhesion‐based photocleavable 2‐nitrobenzyl group. Reproduced with permission.^[^ ^34^ ^]^ Copyright 2004, American Chemical Society. (B) Microscopy images of adhered cells on RGD‐modified SAM with photosensitive 4,5‐dialkoxy 1‐(2‐nitrophenyl)‐ethyl linker before and after UV irradiation. Reproduced with permission.^[^ ^35^ ^]^ Copyright 2011, John Wiley and Sons. (C) Schematic representation of bioligand removal from peptide nanofiber surfaces after exposure to UV light. (D) Representative cell morphologies of on various surfaces (with or without UV irradiation). Reproduced with permission.^[^ ^36^ ^]^ Copyright 2012, American Chemical Society. (E) The chemical structure changes of surface attached RGD peptide with photolabile caging group after irradiation at 351 nm. Reproduced with permission.^[^ ^37^ ^]^ Copyright 2008, American Chemical Society. (F) Photochemical reaction of a surface tethered photo‐caged RGD peptide with nitrobenzyl group. Reproduced with permission.^[^ ^38^ ^]^ Copyright 2008, John Wiley and Sons. (G) Schematic illustration of the using of UCNPs for NIR light‐controlled cell adhesion. (H) Fluorescence images of NIR light‐induced cells release. (I) In‐depth tissue irradiation by covering with pork tissue and the cell release efficiency. Reproduced with permission.^[^ ^39^ ^]^ Copyright 2014, American Chemical Society

Photocleavable molecules, such as 2‐nitrobenzyl groups,^[^ ^22^, ^31^ ^]^ pyrene esters,^[^ ^32^ ^]^ and coumarin ester,^[^ ^33^ ^]^ can undergo chemical bond cleavage upon the irradiation with ultraviolet or near‐infrared (NIR) light. Surface biomolecular modification using photocleavable groups as the linkers may lead to dynamic biointerfaces with photo‐switched bioactivity presentation. Based on this idea, Nakanishi et al. reported a silane coupling agent (1‐(2‐nitrophenyl)ethyl‐5‐trichlorosilylpentanoate, NPE‐TCSP) linked with 2‐nitrobenzyl (a photocleavable group) (Figure 4A).^[^ ^34^ ^]^ Glass slides modified with NPE‐TCSP were covered with bovine serum albumin (BSA) via hydrophobic interaction to obtain an inert surface for cell binding and adhesion. After UV exposure at wavelength of 365 nm, the 2‐nitrobenzyl linkers were cleaved and surface BSA was dissociated, leaving the carboxyl groups capable of binding fibronectin (a cell adhesive protein). This dynamic surface with photo‐triggered bioligand release demonstrated the possibility to spatially control cell adhesion. Taken this idea one step further, del Campo and co‐workers fabricated a dynamic surface using a bioadhesive nitrophenyl‐based photocleavable linker (Figure 4B).^[^ ^35^ ^]^ The photocleavable linker contained an RGD or biotin terminal, a tetraethyleneglycol (TEG) spacer and an interspersed 4,5‐dialkoxy 1‐(2‐nitrophenyl)‐ethyl photolabile group. The biological ligand (RGD or biotin) enabled mediating specific molecular or cell binding. Upon exposure under UV light, the chromophore nitrophenyl group was photocleaved, and biological ligands were effectively removed, finally resulting in a bioinert surface. The authors demonstrated that the RGD‐terminated photolabile group could mediate accurate cell adhesion and ensuing light‐responsive cell detachment from the surfaces. This work is the first example of dynamic biointerfaces capable of photocontrolled release of cell adhesive RGD motifs and detachment of the adhered cells. In a further work, Sur et al. applied this approach for dynamic nanomaterial interfaces by developing a photoresponsive peptide self‐assembly with surface nitrophenyl‐linked RGD peptide (Figure 4C).^[^ ^36^ ^]^ The peptide‐assembled cylindrical nanofiber hydrogels could be used as synthetic ECM with photodynamic controlled bioactivity (Figure 4D).

Apart from photo‐triggered bioligand release, the photocleavable groups can be employed to design photocaged peptides and proteins, which are a set of potential photoswitchable bioligands binding for cells.^[^ ^37^, ^38^, ^40^ ^]^ Photocaged peptides or proteins are initially inactive. Upon light exposure, photocleaving process will lead to a recovery of the caged peptides and proteins, enabling a spatiotemporal activation of biological functions. del Campo's group first employed photocaged concept to inactivate/activate the cell adhesive peptide RGD (Figure 4E).^[^ ^37^ ^]^ They modified an RGD cyclopeptide by using 3‐(4,5‐dimethoxy‐2‐nitrophenyl)‐2‐butyl ester (DMNPB) as the photolabile caging group on the carboxylic acid sidechain of the Asp residue. The DMNPB‐modified RGD cyclopeptide was grafted on silica using a tetra(ethylene glycol) (TEG) spacer. Due to the cell‐repelling property of TEG and the inactivity of DMNPB‐modified RGD, a few adhered cells were discovered on DMNPB‐caged substrates. In contrast, after UV‐triggered RGD uncaging process the dynamic surface showed an in situ site‐selective enhancement of cell adhesion. Similarly, Yuki et al. reported a similar photocaging concept for photocontrolled cell adhesion by introducing 2‐nitrobenzyl group at the amide bond between the Gly and Arg residues (Figure 4F).^[^ ^38^ ^]^ Despite the different sites in the modification of RGD with photolabile caging group, the above two methods were designed to realize the photo‐switched spatial control of cell adhesion. We can imagine further explorations on some potential applications like in situ photo‐patterning of cell arrays and light‐directed cell migration may be readily performed. In fact, in vivo control of bioligand display through subcutaneous implantation of a photocaged hydrogel was also reported.^[^ ^41^ ^]^ Spatial cell patterning in vivo could be achieved on the implanted hydrogel. Moreover, the authors have demonstrated the potential of this strategy to regulate inflammation, fibrous encapsulation, and vascularization in vivo. This study implied the possibility of these photocaged biomaterials for remote control or regulation of tissue repair in vivo.

Recently, it has been recognized that the UV light stimulus process is invasive to cells and its poor penetrability in tissues greatly limited the in vivo applications. In contrast, the NIR light has been proven to have minimal cell damage and enable deeper tissue penetration. In this light, Li et al. fabricated a NIR photocleavable substrate for dynamic control of cell–material interaction (Figure 4G).^[^ ^39^ ^]^ To introduce NIR photocleavage into the system, an upconversion nanoparticle (UCNP) covalently grafted with an RGD‐terminated and 4‐(hydroxymethyl)‐3‐nitrobenzoic acid (ONA) UV photocleavable molecule was employed for material surface engineering. The UCNP materials could absorb NIR (980 nm) and transform them into local UV light, which was subsequently absorbed by photolabile linkers. This procedure resulted in the photocleavage of ONA and the subsequent release of RGD motifs. The results indicated that 70% of the well‐adhered cells could detach from the surface after irradiation with 4 w/cm^2^ NIR light for half an hour (Figure 4H). Moreover, no obvious decrease in cell viability was detected in the cells treated with NIR laser. The authors further demonstrated the possibility of this system for NIR photocontrol of cell adhesion under deep tissues (Figure 4I). Even under a muscle tissue with 4 mm depth, NIR exposure could still trigger and release 50% of the cells, suggesting that this NIR photocleavable system may have a wide range of applications in the regulation of in vivo cell adhesion. To sum up, the photon upconversion strategy reported in this work represents a very promising photocontrol mean for in vivo cell regulation in future tissue engineering and regenerative medicine.

#### 2.1.3 Quinone electrochemical redox reaction

**FIGURE 5:**
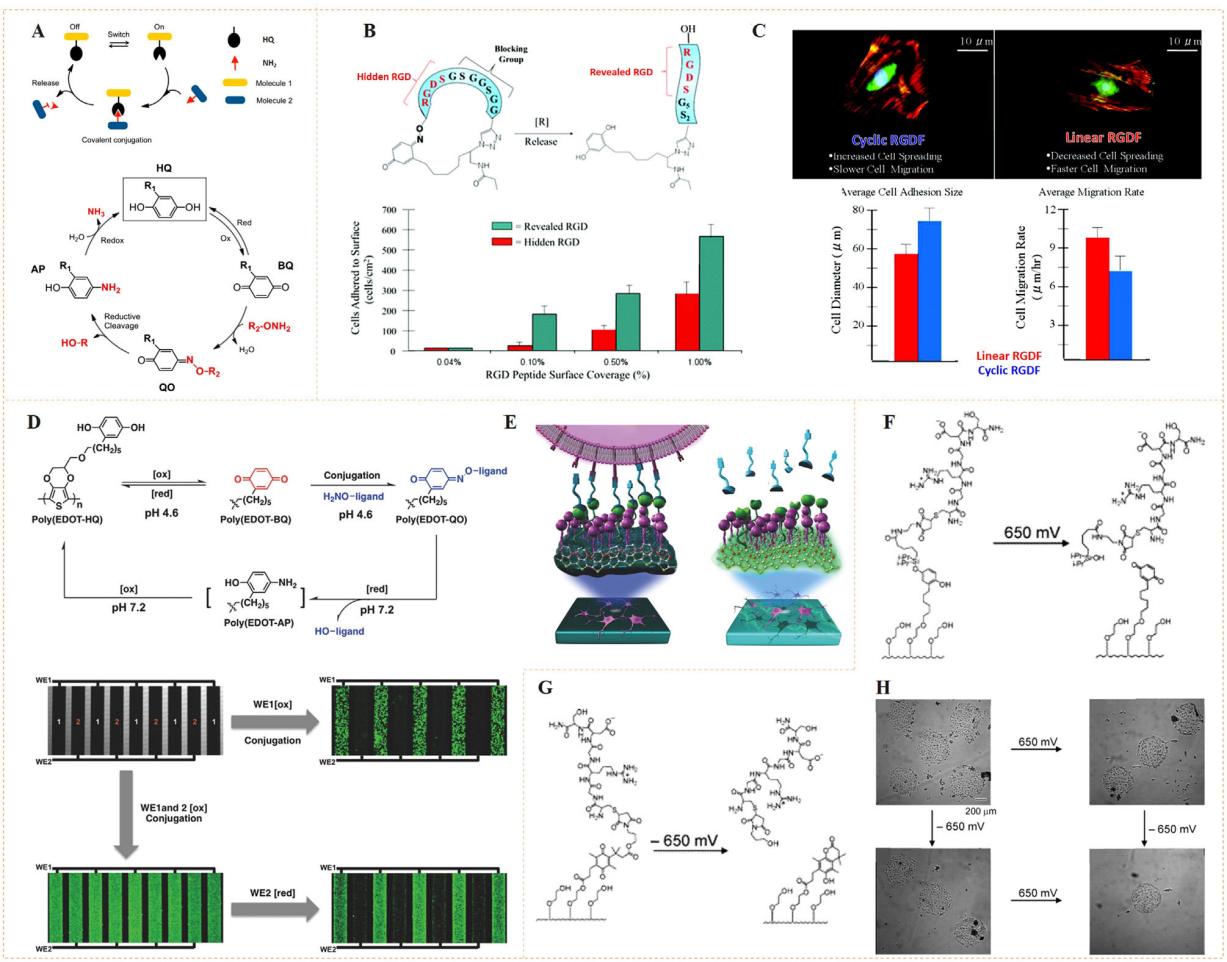
Dynamic presentation of bioligands through electrochemical redox reaction. (A) Schematic illustration of the chemoselective redox‐responsive molecular conjugation and release mechanism based on hydroquinone (HQ). Reproduced with permission.^[^ ^43^ ^]^ Copyright 2014, American Chemical Society. (B) The hide‐and‐reveal ligand strategy based on RGD‐peptide cyclization for controlling cell attachment. Revealed RGD showed significantly higher cell attachment. (C) Fluorescent images of cells on the linear and cyclic RGD surfaces showed different cell adhesion and migration rates. Reproduced with permission.^[^ ^44^ ^]^ Copyright 2011 American Chemical Society. (D) PEDOT‐HQ film enabled the formation/cleavage of oxime linkages for cyclized ligand conjugation, which showed selective immobilization and release of aminooxy‐functionalized fluorescent dyes on PEDOT‐HQ‐coated Au microelectrodes. (E) Schematic illustration of electrical communication and redox‐triggered interactions between neurons and dynamic PEDOT surface. Reproduced with permission.^[^ ^45^ ^]^ Copyright 2008, John Wiley and Sons. (F) Electrochemical oxidation of O‐silyl hydroquinone‐coated substrate gives a benzoquinone with the hydrolysis of the silyl ether and the selective release of RGD ligand linked. (G) Electrochemical reduction of the quinone ester‐coated substrate leads to a cyclization reaction and gives a lactone followed with the release of the RGD ligand linked on the ester part. (H) Selective release of adherent cells under different electrochemical controls. Reproduced with permission.^[^ ^46^ ^]^ Copyright 2006, American Chemical Society

Different from the photon energy used in photochemical cleavage, electrochemical cleavage uses electron energy as nonspontaneous chemical transformation. The electron‐transfer process leads to redox reactions, which may be used to shift the redox state of chemical groups for potential applications. The reversible redox reaction between benzoquinone and hydroquinone (BQ/HQ) and its inherent role in electron and transfer of proton have been extensively studied.^[^ ^20^, ^42^ ^]^ Recent studies found that the BQ/HQ system enables oxime ligation to reversibly immobilize amino‐terminated molecules, since oxime linkage can be cleaved and recovered to the HQ state at a certain reduction potential (Figure 5A).^[^ ^43^ ^]^ With this electrochemical cleavable group, Lamb and Yousaf developed an electrochemical redox‐switchable surface with hide‐and‐reveal dynamic strategy for dynamic control of cell‐binding ligand structure (Figure 5B).^[^ ^44^ ^]^ The dynamic surface was based on a tailored RGD peptide with electroactive HQ, in which the quinone–oxime linkage enables a cyclization of the RGD peptide. Upon applying a noninvasive electrochemical potential, the RGD peptide underwent a structure change from cyclic to linear resulting from the redox‐caused cleavage of oxime linkage. The author found that the cyclic peptide‐functionalized surface showed poor cell adhesion, probably due to the bending structure of cyclic peptide that shielding the RGD motif from cell integrin receptors (Figure 5C). When the electrochemical cleavage of the oxime occurred, the linear RGDS sequence became accessible for cell membrane integrin binding, leading to an electroactive dynamic biointerface capable of electrochemical control of cell adhesion.

The above electrochemical cell regulation has high potential in advanced bioelectronics, such as neural probes, tissue regeneration, and transplantation. Bioelectronic materials require good electrical communication, excellent biocompatibility, and adaptable mechanical properties.^[^ ^47^ ^]^ Inspired by the electrochemical cleavage of quinone–oxime linkage, Yu's group integrated conductive poly(3,4‐ethylenedioxythiophene) (PEDOT) film with electroactive HQ for redox‐switchable surface biofunctionalization (Figure 5D).^[^ ^45^ ^]^ PEDOT has low interfacial impedance, nonspecific‐binding resistance, and flexibility. HQ‐modified PEDOT film, (PEDOT‐HQ), thus possesses excellent electrical response and biocompatibility and enables electroactive biofunctionalization for dynamic control of cell–biomaterial interactions. The authors demonstrated that, an efficient electrical stimulation on the PEDOT‐HQ film could greatly strengthen neurite differentiation and without cell loss. It could also release the neurite cells with high viability and morphological integrity (Figure 5E). The conductivity, biocompatibility, and electrochemically reversible biofunctionalization of this PEDOT‐HQ film have great potential for the fabrication of a dynamic cell–bioelectronicbrk interface.

In addition to the quinone–oxime linkage, there are also other types of electrochemical cleavable systems based on the redox reactions of quinone. Two different quinone derivatives, that is, the quinone ester (QE) and O‐silyl hydroquinone (SHQ), are both electroactive molecules capable of releasing the tethered ligands in reaction to both reductive and oxidative potentials (Figure 5F,G).^[^ ^46^ ^]^ QE can undergo a reduction to produce hydroquinone, which forms a lactone through cyclization reaction to release the eater‐linked bioligand. In contrast, the SHQ group can form a benzoquinone by electrochemical oxidation, followed by silyl ether hydrolysis and subsequently release of the ligand. These two dynamic systems were both able to control bioligand‐mediated specific cell adhesion using electrical stimulations (Figure 5H), expanding the tool kit of current electrochemical conjugation and release strategies for dynamic biointerface fabrication.

#### 2.1.4 Potential‐controlled molecular shielding

**FIGURE 6:**
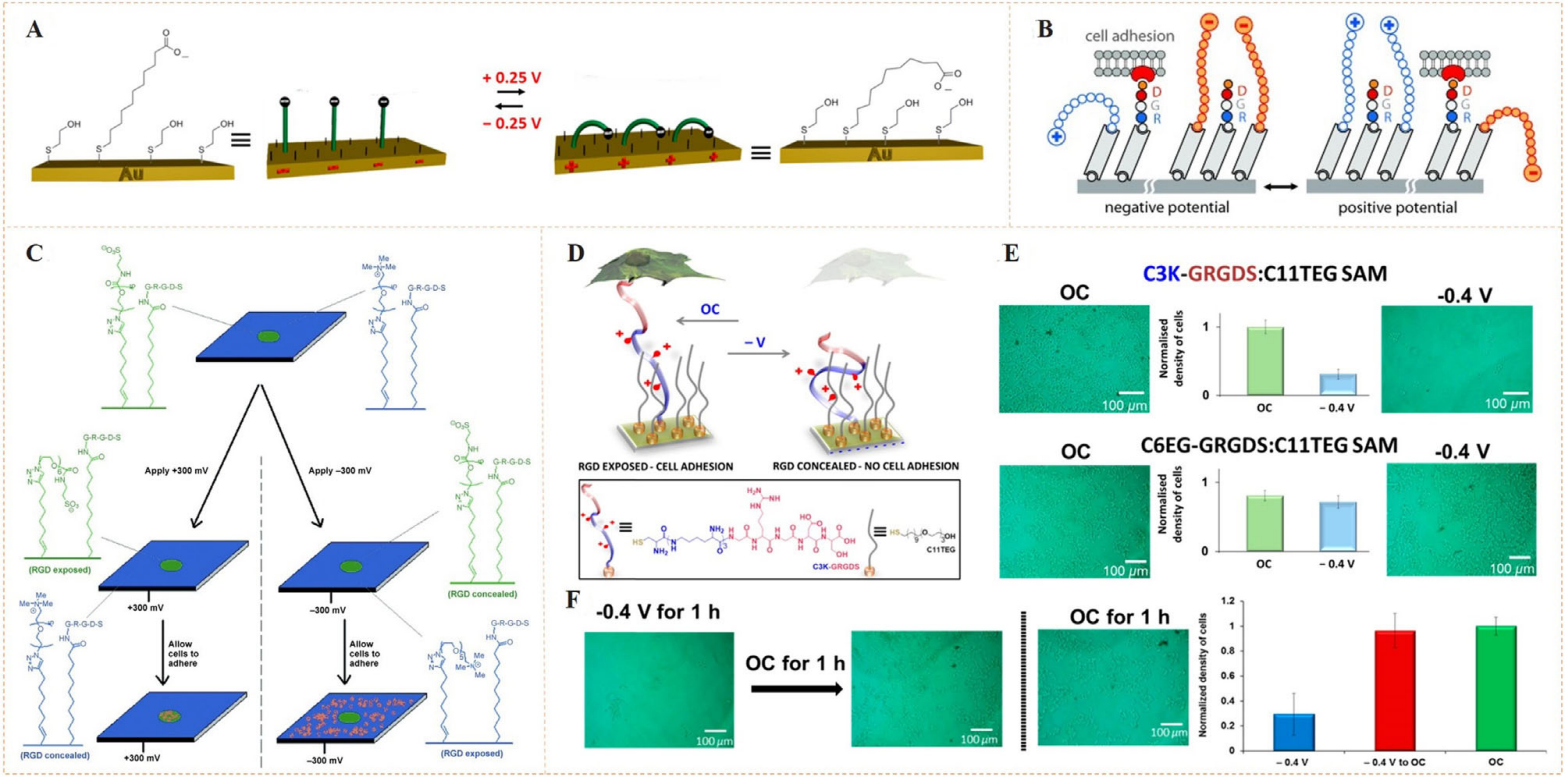
Dynamic presentation of bioligands through potential‐controlled molecular shielding. (A) Schematic illustration of general mechanism of a potential‐controlled molecular shielding. Reproduced with permission.^[^ ^49^ ^]^ Copyright 2013, John Wiley and Sons. (B) The control mechanism of potential‐controlled molecular shielding of the tethered RGD peptides. (C) The molecular design and operation principle of electric switchable surfaces for controlled cell adhesion. Reproduced with permission.^[^ ^51^ ^]^ Copyright 2012 John Wiley and Sons. (D) Schematic of the dynamic RGD oligopeptide SAM for electric control of specific cellular interactions. Under open circuit (OC) conditions (no applied potential), RGD could be exposed for cell adhesion, while a negative potential led to RGD concealing and cell adhesion inhibition. (E) Microscopic images and density of adhered cells on the potential‐responsive C3K‐GRGDS:C11TEG SAM and the control nonresponsive C6EG‐GRGDS:C11TEG SAM. (F) The highly effective transition from a non‐adhesive to cell‐adhesive state on the C3K‐GRGDS:C11TEG SAM surface. Reproduced with permission.^[^ ^52^ ^]^ Copyright 2014, the Royal Society of Chemistry

Similar to the azobenzene isomerization, charged molecules tethered on a conductive surface can also undergo reversible conformational or oriented changes through the simulation of an electrical potential.^[^ ^48^ ^]^ On a surface tethered with charged molecules, different electrical potentials (forward or reverse) can lead to different molecular states (stretching or bending) due to the chemo selective charge adsorption/repulsion effects (Figure 6A).^[^ ^49^ ^]^ Such potential‐controlled changes in molecular conformation have proven to be useful for modulation of biointeractions.^[^ ^49^, ^50^ ^]^ This suggests that a covalent bound bioligand may show different tethering states with accessibility or inaccessibility for the binding of cell membrane receptors under different potential.

Gooding and co‐workers demonstrated the feasibility of using surface tethered bioligand with electro‐switchable bioactivity for the control of cell adhesion.^[^ ^51^ ^]^ They prepared two SAMs that comprised cell repellent oligo(ethylene glycol) (OEG) chains functionalized with different charged moieties (i.e., the anionic sulfonate or the cationic ammonium) at each end (Figure 6B,C). An RGD‐terminated component with a shorter chain was also inserted on the two SAMs, as the cell‐binding model bioligand. If the SAM was placed under a same polarity potential as the charged moiety, the OEG groups showed a stretching state that could conceal the RGD peptides from the cells (i.e., molecular shielding effect). In contrast, if the potential is switched to an opposite polarity, the OEG groups might be blended and the RGD ligands might be exposed allowing cells to recognize them. The two SAMs thus could reversibly regulate cell adhesion upon the reverse of the applied electrical potential, showing electro‐switchable control of cell behaviors. Mendes and co‐workers then reported a similar work, while in their work the RGD ligand was tethered on Au surface through a lysine‐containing (positive charged) oligopeptide linker as the electric‐switching unit (Figure 6D–F).^[^ ^52^ ^]^ The conformational changes of the oligopeptide from stretching to contracting states can be employed to selectively expose or conceal the RGD, resulting in electrodynamic regulation of cell adhesion.

Zhang et al. constructed an intriguing potential‐responsive SAM‐based biointerface capable of controlling cell adhesion and stem cell differentiation.^[^ ^53^ ^]^ Similar to the mechanism of Gooding's work, reversibly modulated cell adhesion could be achieved by applying potentials of different polarities to the SAM electrode surface. Using electric potentials, cells might be detached from the electrode as whole clusters with varying shapes. More crucially, the potential‐triggered mechanism may affect cell shape during adhesion, influencing stem cell development. The work indicated that the potential‐controlled molecular shielding effect could be flexibly controlled by the intensity of electric potential, which would facilitate the development of dynamic systems with tunable bioligand densities for cell receptors. Therefore, this dynamic strategy shows great potential to adapt the complexity of in vivo biochemical cues, for example, the spatiotemporal presentation.

#### 2.1.5 Dichalcogenide molecular exchange

**FIGURE 7:**
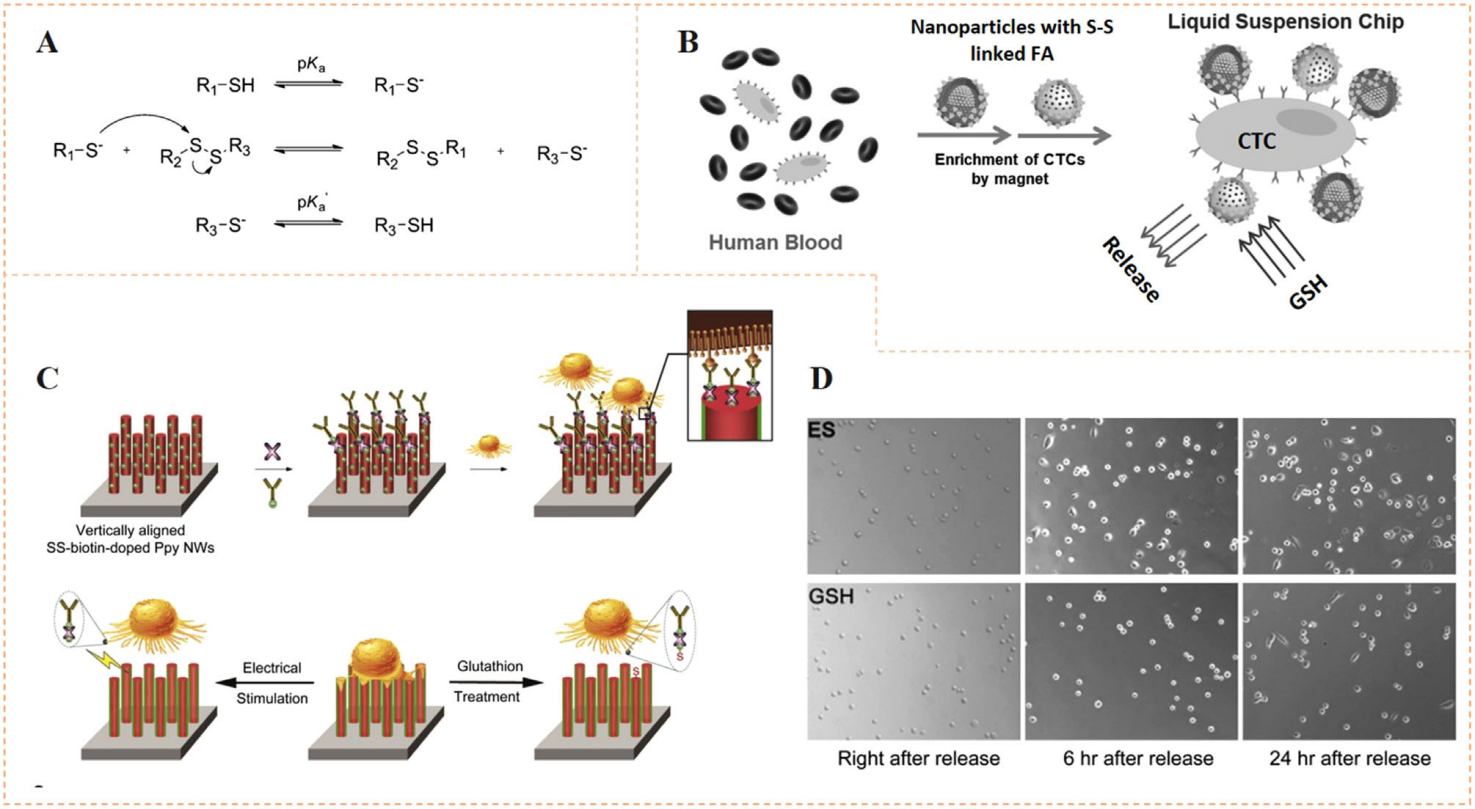
Dynamic presentation of bioligands through disulfide molecular exchange. (A) The molecular exchange mechanism of disulfide with another disulfide or thiol groups. (B) The microsphere suspension chip with S‐S‐bound FA for quick capture and GSH‐triggered release of CTCs from human blood. Reproduced with permission.^[^ ^55b^ ^]^ Copyright 2015, John Wiley and Sons. (C) The anti‐EpCAM‐immobilized SS‐biotin‐doped PPy nanowires for capturing, releasing, and quantifying cancer cells in situ. (D) Cell spreading and proliferation at various time points after ES‐ or GSH‐mediated release at various time periods. Reproduced with permission.^[^ ^56^ ^]^ Copyright 2014, Elsevier B.V

Dichalcogenides such as disulfides, diselenides, or mixed sulfur–selenium bonds have been widely used for the preparation of dynamic materials due to their reversibility.^[^ ^54^ ^]^ Typically, a disulfide bond can easily occur molecular exchanging with another disulfide or thiol groups (Figure 7A). Moreover, external stimuli like catalysts, light irradiation, and high temperature can accelerate the exchange reaction. Therefore, the introduction of dichalcogenide bonds is also a potential means to construct dynamic biointerfaces with reversible bioligand presentation and controlled cell recognition.^[^ ^55^ ^]^ For example, Li et al. introduced cancer‐targeting folic acid (FA) on magnetic microspheres for targeted capture and release of HeLa cells by applying reversible disulfide bond (Figure 7B).^[^ ^55b^ ^]^ The dynamic system could capture 95% of HeLa cells within 15 min, but did not capture HEK 293T cells at the same time. In addition, glutathione (GSH) could be used to cut disulfide and separate the captured tumor cells. The cell release efficiency was more than 90%. Likewise, Hong et al. used disulfide (SS)‐biotin doped polypyrrole nanowires (Ppy‐NWs) to construct a multifunctional system for in situ capturing, releasing, and quantifying circulating tumor cells (CTCs) (Figure 7C).^[^ ^56^ ^]^ The surface SS‐biotin could bind with streptavidin and connect with a biotinylated anti‐epithelial cell adhesion molecule (anti‐EpCAM), showing specific recognition of EpCAM‐positive cancer cells. The captured cells could be released by GSH due to the molecular swapping capacity of disulfide linkers. In fact, this work also involved a noncovalent strategy for dynamic bioligand conjugation. External electrical stimulation (ES) could reverse the surface charge of Ppy, which could also be helpful for the release of surface doped biotin and also the captured cells. Detailed mechanism of electroactive Ppy will be discussed in Section 2.2.3. With this multi‐responsiveness, the dynamic system showed excellent CTCs capture/release. The efficiency for capture of breast cancer cells (MDA‐MB‐468) with high expression of EpCAM was greater than 93%. Especially in relatively low concentrations (e.g., 10–20 cells/ml), the 98.5% mean capture rate, and 95.69% release efficiency could be reached using GSH exchange. Simultaneously, the nonspecific capture of leukocytes in the captured cell solution was significantly reduced. Moreover, reseeding of the released cells on plates demonstrated that the treatment with GSH or ES showed no significant invasiveness to the cells (Figure 7D).

These successful records by using dynamic disulfide bonds for bioligand conjugations provide a hint that the diselenide or sulfur–selenium bonds with similar molecular exchangeability may get the same effects. In the past decade, dynamic diselenide bonds have been extensively studied.^[^ ^57^ ^]^ These emerging dichalcogenides have been proven to possess some unique properties, for example, visible light induced exchange reaction.^[^ ^58^ ^]^ We believe that novel dynamic biointerfaces based on diselenide or sulfur–selenium bonds will be developed in a near future.

#### 2.1.6 Phenylboronate ester exchange

Phenylboronate ester, formed by condensation of phenylboronic acid (PBA) and *cis*‐diol/ catechol, is a type of dynamic covalent bonds which was extensively applied to the constitution of molecular receptors.^[^ ^59^ ^]^ Phenylboronate esters are formed in alkaline environment and may break under acidic condition, showing pH sensitivity.^[^ ^60^ ^]^ The phenylboronate–catechol bonds were also found with potential sensitivity through electrochemical oxidation process.^[^ ^61^ ^]^ It is worth mentioning that if phenylboronate ester is incubated with another *cis*‐diol compound, competitive reaction may occur with a diol molecular exchanging process to form a new ester bond. Since saccharides are rich in *cis*‐diol groups, phenylboronate esters thus can exhibit sugar sensitivity, a relatively biocompatible molecular dynamic.^[^ ^62^ ^]^ In view of this, PBA has been widely used in sugar detection, glycoprotein purification, biosensing, controlled drug release, dynamic material fabrication, and so on.^[^ ^63^ ^]^ Since cells can specifically express glycoproteins on the membranes, PBA‐functionalized substrate have been fabricated for capturing and releasing specific cells or bacteria through the reversible binding between PBAs and the membrane glycans.^[^ ^61^, ^64^ ^]^

**FIGURE 8:**
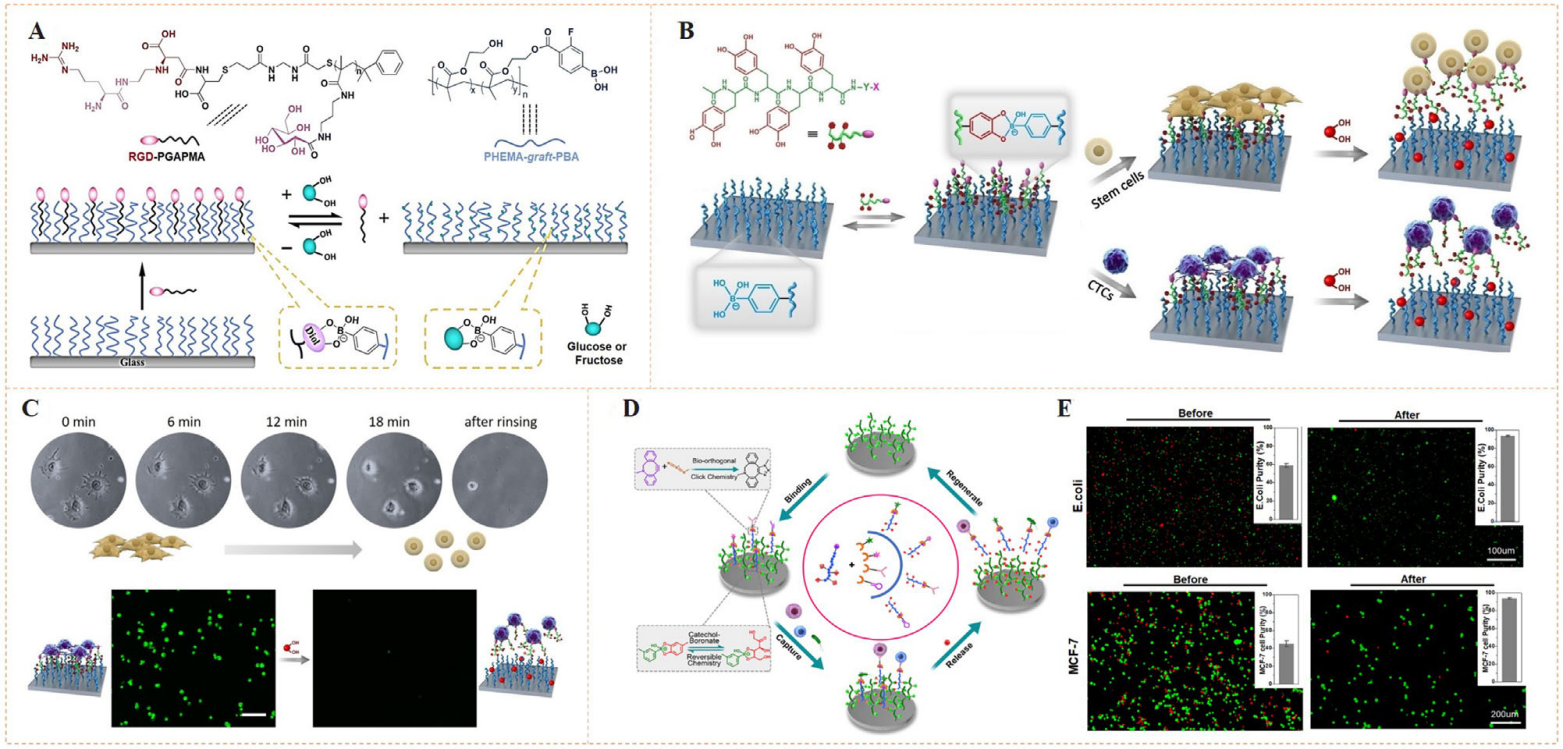
Dynamic presentation of bioligands through reversible phenylboronate covalent bonds. (A) The molecular exchange mechanism of phenylboronic acid‐*cis*‐diol complex with sugar molecules. Reproduced with permission.^[^ ^65^ ^]^ Copyright 2014, American Chemical Society. (B) Schematic illustration of the phenylboronate‐based mussel‐inspired dynamic biointerface with different cell‐binding ligands for control of cell adhesion and selective cell capture/release. (C) Dynamic cell adhesion and cancer cell isolation could be achieved by addition of sugar into the system. Reproduced with permission.^[^ ^66^ ^]^ Copyright 2015, John Wiley and Sons. (D,E) Schematic of upgraded mussel‐inspired dynamic biointerface with optional and diversified bioactivities for different pathogenic cell capture and release. Reproduced with permission.^[^ ^68^ ^]^ Copyright 2020, Elsevier B.V

The sugar‐sensitive molecular exchangeability of phenylboronate ester bonds also allows reversible biomolecular conjugation. Pan's et al. reported, for the first time, the application of phenylboronate ester bonds with sugar sensitivity for dynamic recognition of bioligands and reversible modulation of cell activities (Figure 8A).^[^ ^65^ ^]^ The fabricated biointerface was created by grafting RGD‐conjugated poly(3‐gluconamidopropyl methacrylamide) (RGD‐PGAPMA) glycopolymer and poly(hydroxyethyl methacrylate‐PBA) (PHEMA‐PBA) molecular brushes on glass substrate. The RGD‐PGAPMA glycopolymer can covalently bind on the PHEMA‐g‐PBA‐grafted biointerface through multi‐covalent phenylboronate ester bonds. The authors demonstrated that after adding the medium with *cis*‐diol‐containing sugars (e.g., glucose or fructose) the bound RGD‐PGAPMA could be released that could exchange with PGAPMA glycopolymer. With this dynamic molecular mechanism, cell could reversible adhesion and detachment on a PBA‐containing biointerface which could be readily regulated by imitating a natural biofeedback system (i.e., human glycemic volatility).

The above study suggested that a long linear glycopolymer capable of forming numerous phenylboronate ester linkages is required to achieve a stable but reversible molecular interaction. The artificial chemicals (i.e., polymers or chemical linkers), however, are potentially nonbiocompatible and detrimental to cells. Given this, Pan's group further designed a set of mussel biomimetic peptides for reversible bioligand presentation (Figure 8B).^[^ ^66^ ^]^ As a natural catecholic amino acid, 3,4‐dihydroxy‐l‐phenylalanine (DOPA) is discovered in high concentrations in mussel‐secreted proteins. Since catechol can also form reversible phenylboronate ester, the authors designed a biomimetic peptides (DOPA)_4_‐**Y**‐**X**, which consists of a flexible bioactive sequence X at the C‐terminus, a nonbioactive spacer sequence Y, and a (DOPA)_4_ at the N‐terminus. The (DOPA)_4_‐**Y**‐**X** peptides could bind with PBA‐grafted surface and leave the exposed X sequence for cell recognition. Upon the addition of excessive sugars (e.g., glucose or fructose), the release process of (DOPA)_4_‐**Y**‐**X** peptide occurred, exhibiting a sugar‐responsive surface bioactivity. By binding two types of cell‐binding sequences (e.g., RGD and tumor‐targeting WxEAAYQrFL^[^ ^67^ ^]^), the dynamic surface could be endowed with different potentials. For example, in addition to the reverisble manipulation of stem cell adhesion behaviors, tumor cells can also be selectively captured and released (Figure 8C). The results indicated the versatility of this dynamic biointerface in biomedicine.

Taking this work one step further, the same group reported another mussel‐inspired dynamic biointerface by the combination of catechol‐boronate with bioorthogonal click chemistry to obtain optional and diversified bioactivities (Figure 8D).^[^ ^68^ ^]^ To verify diverse bioligands binding on the surface, a tetra DOPA mussel‐inspired peptide sequence and clickable group dibenzocyclooctyne (DBCO) was synthesized. A set of cell‐binding ligands (e.g., saccharide, peptides, and DNA aptamers), functionalized with the DBCO complementary group azido (N_3_), was utilized as the bioactive sources. The clickable mussel‐inspired peptide was able to dynamically attach on PBA‐grafted substrates through the reversible phenylboronate‐catechol linkages, leaving the terminal DBCO groups for grafting of N_3_‐functionalized bioligands via bioorthogonal click reaction. This design resulted in dynamic biointerfaces with diverse bioactivities, capable of reversible recognition, efficient isolation, and purification of different cells including microorganisms (Figure 8E). This strategy may have great potential to modulate a number of interactions between cells and biomaterials, showing more widespread applications in biomedical science.

#### 2.1.7 Imine exchange and acidolysis

The imine is a class of specific groups or chemical molecules that possess the carbon/nitrogen double bond (─C ═ N─). Imines are formed through condensation reaction of carbonyl compounds (e.g., aldehydes, ketones) and ammonia or primary amines, routinely known as ‘‘Schiff's bases.”^[^ ^69^ ^]^ Imines are capable of participating in three types of reversible reactions: hydrolysis, exchange, and metathesis. The dynamic imine bonds have emerged as a diverse tool in chemical synthesis, drug screening, and biomedical engineering.^[^ ^70^ ^]^ Among currently developed imines, the benzoic‐imine has drawn increasing concerns in the field of biomaterials, due to its mild acid cleavability with a critical pH value at 6.5.^[^ ^71^ ^]^ As is known, physiological processes commonly involve pH changes in both the inside of cells and surrounding ECM environment. Therefore, the benzoic‐imine bonds with mild acid sensitivity are potentially applicable for fabricating dynamic biointerfaces and controllable cell adhesion and growth behaviors.

**FIGURE 9:**
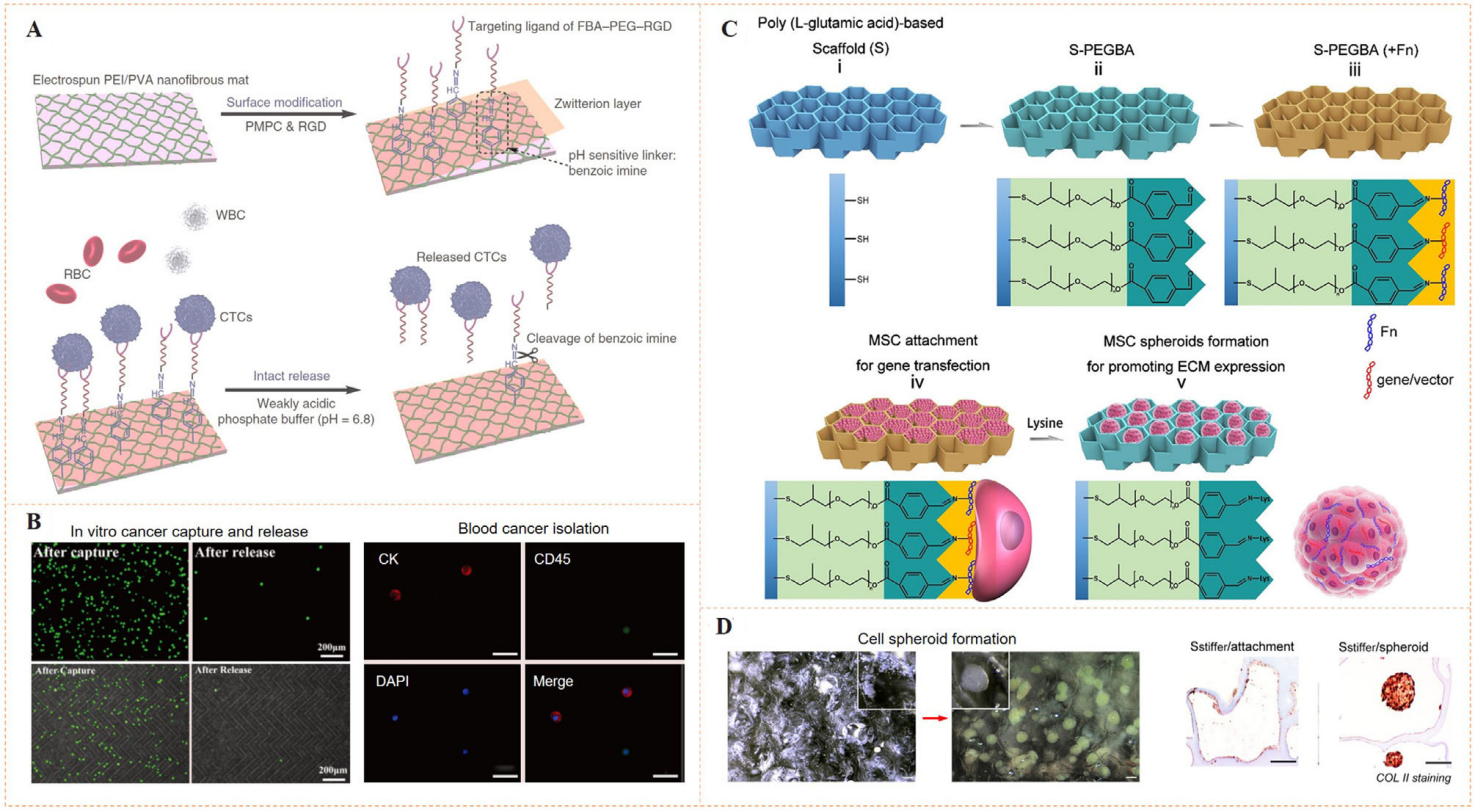
(A) Schematic illustration of electrospun nanofibers film with zwitterionic polymer and RGD functionalization through the benzoic‐imine bond for specific capture and intact release of cancer cells. (B) Cancer cell capture and release on the dynamic benzoic‐imine‐bound RGD surface in vitro and in vivo. Reproduced with permission.^[^ ^72^ ^]^ Copyright 2018, Future Medicine Ltd. (C) Design of the porous scaffold with benzoic‐imine‐linked bioligands for in situ formation of cells spheroids. (D) The formation of cell spheroid on the porous scaffold after lysine treatment. Reproduced with permission.^[^ ^73^ ^]^ Copyright 2014, American Chemical Society

Xiao et al. reported a electrospun polyethylenimine/poly(vinyl alcohol) nanofibers film, which was modified with poly(2‐methacryloyloxyethyl phosphorylcholine) zwitterionic polymer and functionalized with RGD peptide via the mildly acid‐sensitive benzoic‐imine bond (Figure 9A).^[^ ^72^ ^]^ The antifouling ability of zwitterionic polymers and the pH responsiveness of benzoic imine‐linked RGD peptide synergistically led to specific capture integrin highly expressed tumor cells and intact release of them under weakly acidic buffer (pH = 6.8) (Figure 9B). A microfluidic chip coated with this film also showed excellent cell capture and release, and it could be used for isolation of blood cancer cells. The authors also checked the viability of the released cell, revealed that the weakly acidic treatment almost causes no damage to tumor cells. Therefore, the developed pH‐responsive dynamic surface shows the potential in cell‐based disease diagnosis.

Besides macroscopical surface, benzoic‐imine‐mediated dynamic biointerface might also be created on the inner surfaces of macroporous materials, and these miniaturized dynamic surfaces may show unique effects on cell behaviors. For instance, Zhang et al. reported a poly (l‐glutamic acid) (PLGA)‐based porous scaffold with tunable inner surfaces and benzoic‐imine‐linked bioligands (Figure 9C).^[^ ^73^ ^]^ The porous structure facilitated the formation of independent cell community, while the release of bioligands via molecular exchange led to cell‐scaffold detachment and in situ formation of cell spheroids. In this work, fibronectin (Fn) and plasmid DNA encoding transforming growth factor‐β1 (pDNA‐TGF‐β1) were used as the bioligands, for enhancement of cell adhesion and efficient surface‐mediated gene transfection, respectively. After lysine treatment (i.e., imine exchange), the mesenchymal stem cell (MSC) spheroids formed with extensive cell–cell interaction and upregulated TGF‐β1 protein expression with a prolonged term. The MSC spheroids with enhanced chondrogenesis could be directly implanted into cartilage defects for cartilage regeneration (Figure 9D). The work demonstrated the great potential of imine‐mediated dynamic biointerfaces for functionalized cell harvesting, which can be used as cell therapy in tissue engineering.

#### 2.1.8 Enzymolysis

Enzymes mostly are proteins with catalytic abilities crucial to metabolic processes and some biochemical reactions in living systems. The roles of enzymes go beyond the catalytic synthesis of biomolecules, they also work to degrade redundant bioproducts to balance the metabolism through enzymolysis. The enzymolysis of a biomolecule commonly leads to the change or loss of its biological functions. Thus, enzymolysis can be used to regulate the bioactivity of target biomolecules.

**FIGURE 10:**
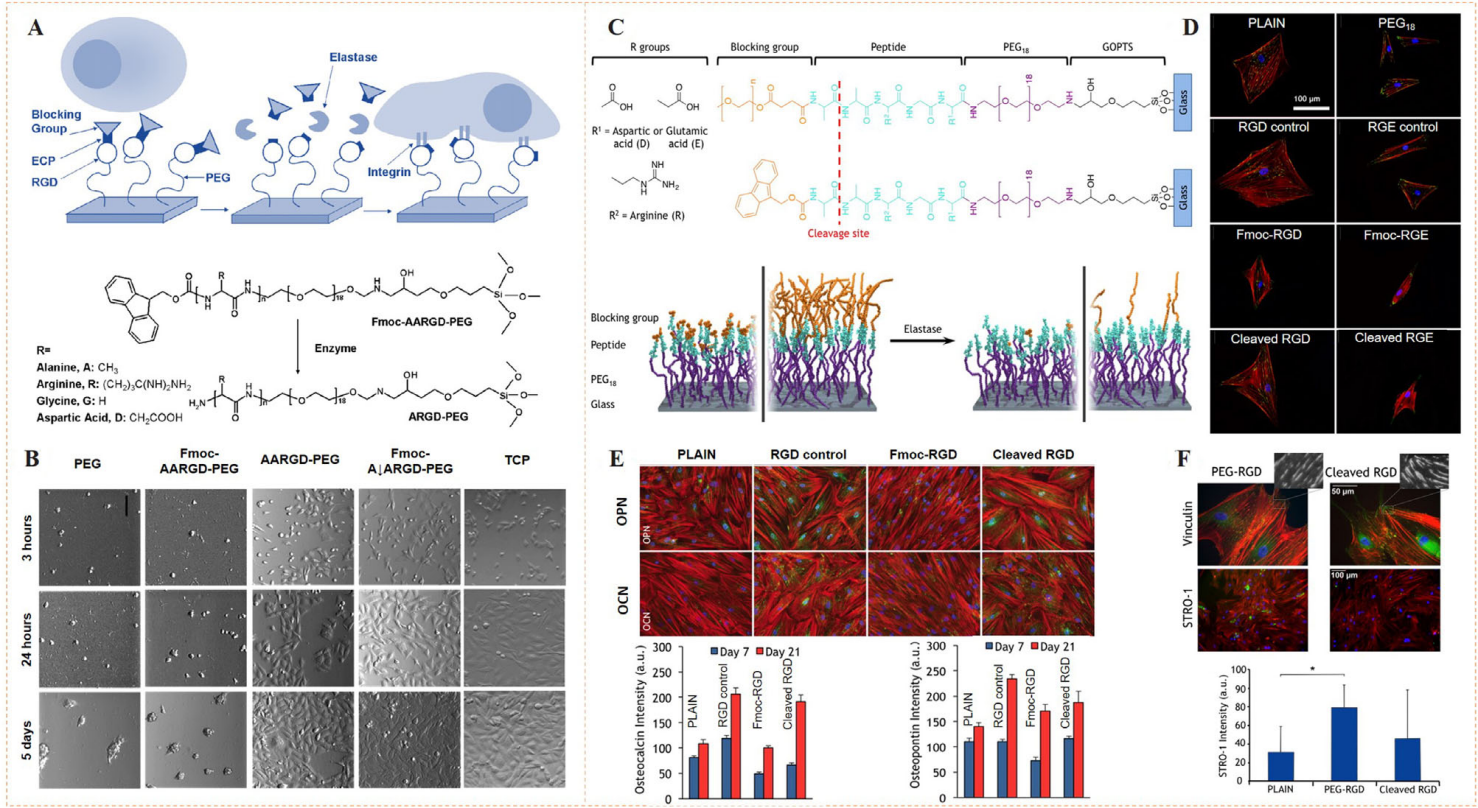
Enzyme‐responsive systems for dynamic bioligand presentation. (A) RGD‐PEG‐linked monolayer with Fmoc blocking groups enabled elastase activation for cell recognition and adhesion. (B) Micrographs of osteoblasts on surfaces with PEG, untreated Fmoc‐AARGD‐PEG, enzyme‐treated Fmoc‐A↓ARGD‐PEG, positive control ARGD‐PEG, and tissue culture polystyrene (TCPS) surfaces. Reproduced with permission.^[^ ^74^ ^]^ Copyright 2009, American Chemical Society. (C) Schematic representation of the Fmoc and PEG blocked RGD surfaces with the elastase cleavage site (dialanine). (D) Dynamic control of stem cell adhesion and tension on different surfaces. (E) Stem cell growth and differentiation on different surfaces. (F) Analysis of stem cell adhesion and differentiation on PEG blocked surfaces. Reproduced with permission.^[^ ^75^ ^]^ Copyright 2016, American Chemical Society

Ulijn and co‐workers pioneered the use of enzyme‐specific peptides for the fabrication of enzyme‐responsive biomaterials with enzymolysis‐triggered ligand activation.^[^ ^74^, ^76^ ^]^ They synthesized an RGD peptide‐functionalized substrate with the terminal R (Arg) amino acid capped with a blocking group. The blocking group is enzyme responsive. Enzyme could result in a cleavage of the blocking group, uncovering the RGD peptide for cell binding. The substrate thus could display enzyme‐responsive dynamic bioactivity and an enzyme‐triggered cell adhesion (Figure 10A,B). Recently, such kind of dynamic surfaces with enzyme‐response ability were further developed for the administration of adhesion/cytoskeletal balance to maintain stem cell cultures. Previous study demonstrated that changes in cell adhesion can be used to affect cell differentiation.^[^ ^77^ ^]^ In view of this, the authors developed an enzyme‐activable dynamic surface using different caging groups (polyethylene glycol and Fmoc).^[^ ^75^ ^]^ In the system, the caged molecule and hidden active molecule (RGD) had discrete and controllable biological activities. (Figure 10C–F). Enzymolysis‐caused cleavage of caging groups led to different effects on cell adhesion. The results showed that on the dynamic surface the adhered cell could be subtly altered by enzyme. As a result, it enabled to turn the state of stem cells from growth to differentiating. This work tactfully employed the specificity of enzyme to trigger minor change in cell adhesion and investigated the adhesion‐regulated stem cell differentiation, providing a new means to precisely control stem cell behaviors for uses in regenerative medicine. Inspired by this work, we expect that diverse enzyme‐responsive systems could be developed in the future to precisely control cell behavior to adapt specific physiological and pathological environments.

#### 2.1.9 Reversible metal coordination

Coordinative interactions widely exist in living systems (e.g., chlorophyll, vitamin metalloprotein), which are crucial to many physiological processes including enzymatic reactions, metabolism, oxygen transport and storage.^[^ ^78^ ^]^ Coordination is a type of alternate covalent bond, relying on the interactions between the central atom (commonly metal ion) and the ligands by sharing electron pairs. Coordination bonds are relatively dynamic compared with typical covalent bonds. The cleavage of a coordinative bond is feasible through ligand exchange or chemical reactions. This property thus endows coordination interactions with reversibility that allows dynamic bioligand binding.^[^ ^79^ ^]^

**FIGURE 11:**
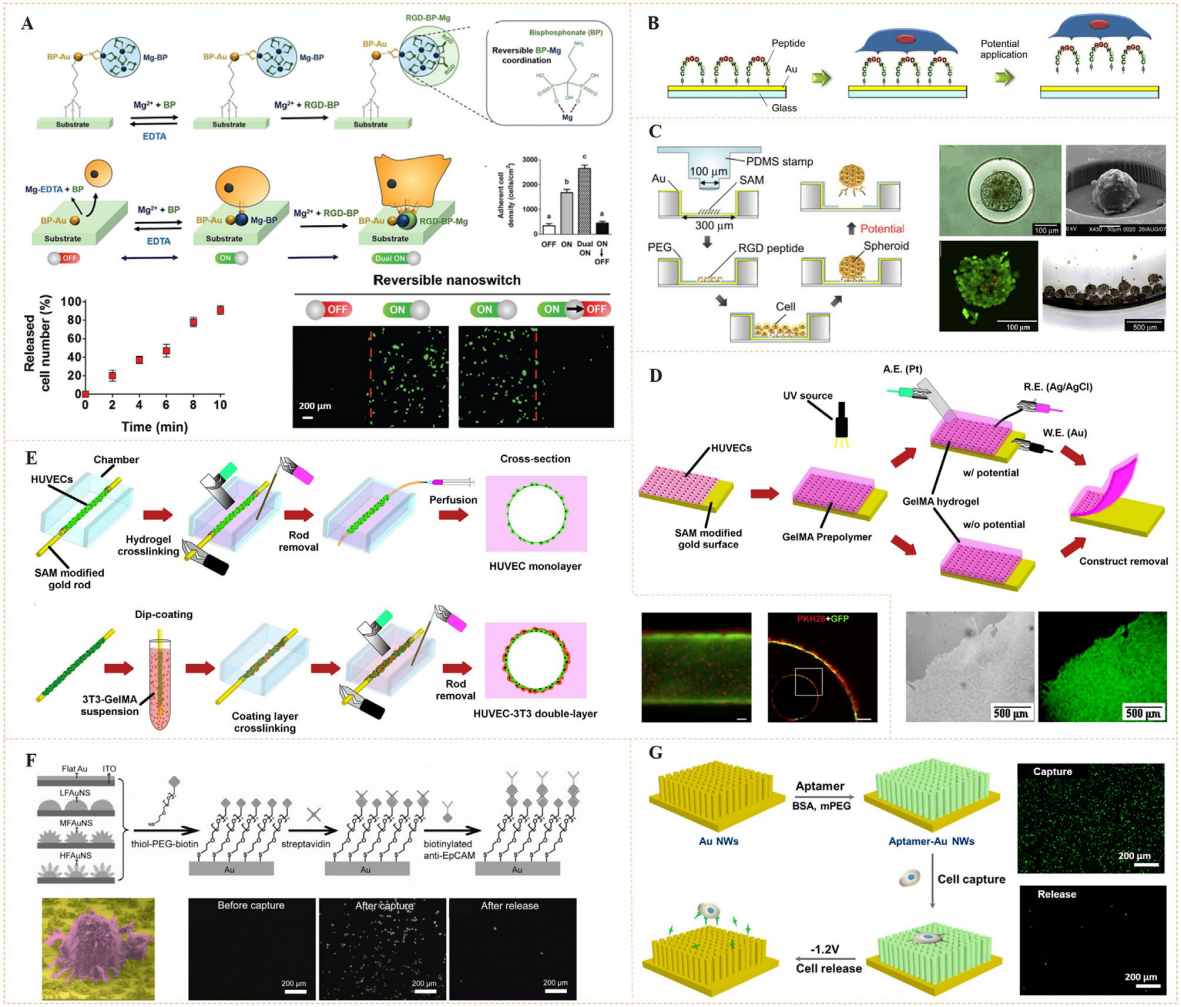
Coordination‐mediated dynamic bioligand presentation. (A) In situ reversible nanoswitching of RGD through reversible Mg^2+^‐bisphosphonate (BP) coordination for regulation of in vivo cell adhesion and release. Reproduced with permission.^[^ ^81^ ^]^ Copyright 2018, John Wiley and Sons. (B) Electrical potential‐responsive RGD release for dynamic control of cell adhesion. Reproduced with permission.^[^ ^82^ ^]^ Copyright 2010, Elsevier B.V. (C) The spheroid culture chip consisted of cylindrical cavities modified with RGD‐capped alkanethiol. Reproduced with permission.^[^ ^83^ ^]^ Copyright 2009, Elsevier B.V. (D) Electrical potential‐triggered cell sheet detachment. (E) Micrometric RGD‐alkanethiol‐coated gold rods enveloped with cells for the electrical potential‐induced cell tube detachment. Reproduced with permission.^[^ ^84^ ^]^ Copyright 2010, Elsevier B.V. (F) Anti‐EpCAM‐modified surface through Au‐S bond for specific recognition of targeted cancer cells and electrical potential‐induced cell detachment. Reproduced with permission.^[^ ^85^ ^]^ Copyright 2013, John Wiley and Sons. (G) The dynamic process of capturing and releasing cancer cells on the aptamer‐modified AuNWs. Reproduced with permission.^[^ ^86^ ^]^ Copyright 2017, American Chemical Society

To fabricate metal coordination‐mediated dynamic biointerface, biocompatible metal ions and adaptable bioligands must be intelligently selected. Magnesium (Mg) is an important element in living systems. It can act as a cofactor involved in a variety of biochemical reactions including energy production, oxidative phosphorylation, and glycolysis. Mg^2+^ has been proven to be a pro‐adhesive cation capable of coordinating and activating metal‐ion‐dependent adhesion sites in integrins.^[^ ^80^ ^]^ In this context, Bian and co‐workers reported a dynamic biointerface that can reversibly switch RGD presentation through the interaction of Mg^2+^ and bisphosphonate (BP) ligands (Figure 11).^[^ ^81^ ^]^ In this work, BP‐bound gold nanoparticles (BP‐AuNP) were first prepared on gold‐plated substrates to initiate in situ self‐assembly of Mg‐BP compound with cell adhesive ability via the coordination of Mg^2+^‐BP. Other bioligand part (i.e., the cell adhesive peptide RGD) was additionally assembled on the substrate by in situ co‐assembly to form a double bioactive RGD‐BP‐Mg^2+^‐BP nanocomposite. Due to the ligand exchangeability of coordination, ethylenediamine tetraacetic acid (EDTA) can be used to decompose Mg^2+^‐BP complex, leading to the release of bioactivities (i.e., the RGD peptide and Mg^2+^) from the surface and subsequently cell detachment. The author also investigated the in vivo effect on dynamic control of cell adhesion. After subcutaneous implantation of the (BP‐AuNP)‐grafted substrates in mice model followed by injection of BP and Mg^2+^, EDTA or RGD‐BP and Mg^2+^ to the mice, the Mg‐BP assembly (“on”), reversible Mg‐BP disassembly (“on→off”) and RGD‐BP‐Mg^2+^ self‐assembly (“dual open”) could be observed in sequence. The RGD‐BP‐Mg^2+^‐BP dual open model was found to be able to significantly regulate and promote cell adhesion and diffusion in vivo. This work perfectly confirmed that metal‐bioligand coordination could be employed for dynamic bioligand presentation, and the resultant sophisticated dynamic biomaterials could regulate not only in vitro cell activities but also in vivo tissue responses.

Besides the ligand‐exchange means, metal coordination may also be able to respond to electrochemical redox reaction. For example, gold‐thiolate bond (Au‐S) can be reductively broken by introducing a negative electrical potential. Using this property, Fukuda and co‐workers have done a series of work for electrochemical desorption of bioligands and detachment of the surface adhered cells.^[^ ^82^, ^83^, ^84^, ^87^ ^]^ Generally, their approach was based on a gold substrate with thiol‐mediated ligand conjugation (Figure 11B).^[^ ^82^ ^]^ The thiol‐ligands could be an RGD‐capped alkanethiol or an RGD‐containing oligopeptide. In the circumstance, cells may adhere to the surfaces. When the substrate was applied with a negative electrical potential, the cells were rapidly released along with the reduction‐caused desorption of the thiol‐ligands. By using this cell detachment approach to different gold surfaces, cells with different geometric structures including cell spheroid (Figure 11C),^[^ ^83^ ^]^ cell sheet (Figure 11D),^[^ ^84^ ^]^ and cell tube (Figure 11E)^[^ ^84^ ^]^ can be readily obtained. This simple but efficient technique could be potentially used as a fundamental tool in cell‐based transplantation for the regeneration of tissues and organs.

Wang and co‐workers further developed this method for catching and releasing the tumor cell (Figure 11F).^[^ ^85^ ^]^ To increase the capture efficiency, gold substrates with fractal nanostructures were prepared. The nanostructural substrates were coated with anti‐EpCAM through thioglycobiotin streptavidin interactions. The authors found that the morphology of gold nanoparticles had a significant impact on capture efficiency of tumor cells. Compared with the flat nanoparticles, the higher fractal gold nanostructures (HFAuNS) showed better capture performance. After 45 min incubation, the capture efficiency of HFAuNS interface was 62 ± 12% for MCF‐7 cells, but the value was only 3 ± 1% for flat Au surface. The reduction of sulfur–gold bond using negative electrical potential could release the captured cells. After exposure under a −1.2 V electric field for 5 min, about 98% of cells captured on the biointerface could be released, and the cell survival rate was more than 98%. In a similar work, Zhai et al. reported dynamic arrays of aptamer‐modified gold nanowires (AuNWs) through Au‐S bonds (Figure 11G).^[^ ^86^ ^]^ The AuNW substrate showed significantly improved tumor cell capture efficiency and rapid electrochemical cell detachment. These studies verified that the electrochemical‐responsive Au‐S coordination is a promising strategy for dynamic bioligand presentation, and the developed platform has great potential in cell‐based disease diagnosis and regenerative medicine.

### 2.2 Reversible noncovalent strategies

#### 2.2.1 Host–guest chemical recognition

Host–guest supramolecular chemistry was usually dominated by macrocyclic host molecules (e.g., cyclodextrins, cucurbiturils, crown ethers, cyclophanes, calixarenes).^[^ ^88^ ^]^ The guest molecules commonly have both a suitable size or shape and functional groups complementary to the macrocyclic hosts, thus allowing for specific recognition between the two parts. Host–guest chemistry has shown great promise in organic synthesis, catalysis, molecular assembly, drug targeting, biochemical analysis, and material design, encompassing almost all disciplines like chemistry, physics, biology, and medicine.^[^ ^89^ ^]^ The controllability of bioligand binding could be improved through host–guest chemistry due to molecular recognition‐mediated reversible interactions,^[^ ^89f^ ^]^ thus showing the potential as a reversible molecular strategy for dynamic bioligand presentation.

**FIGURE 12:**
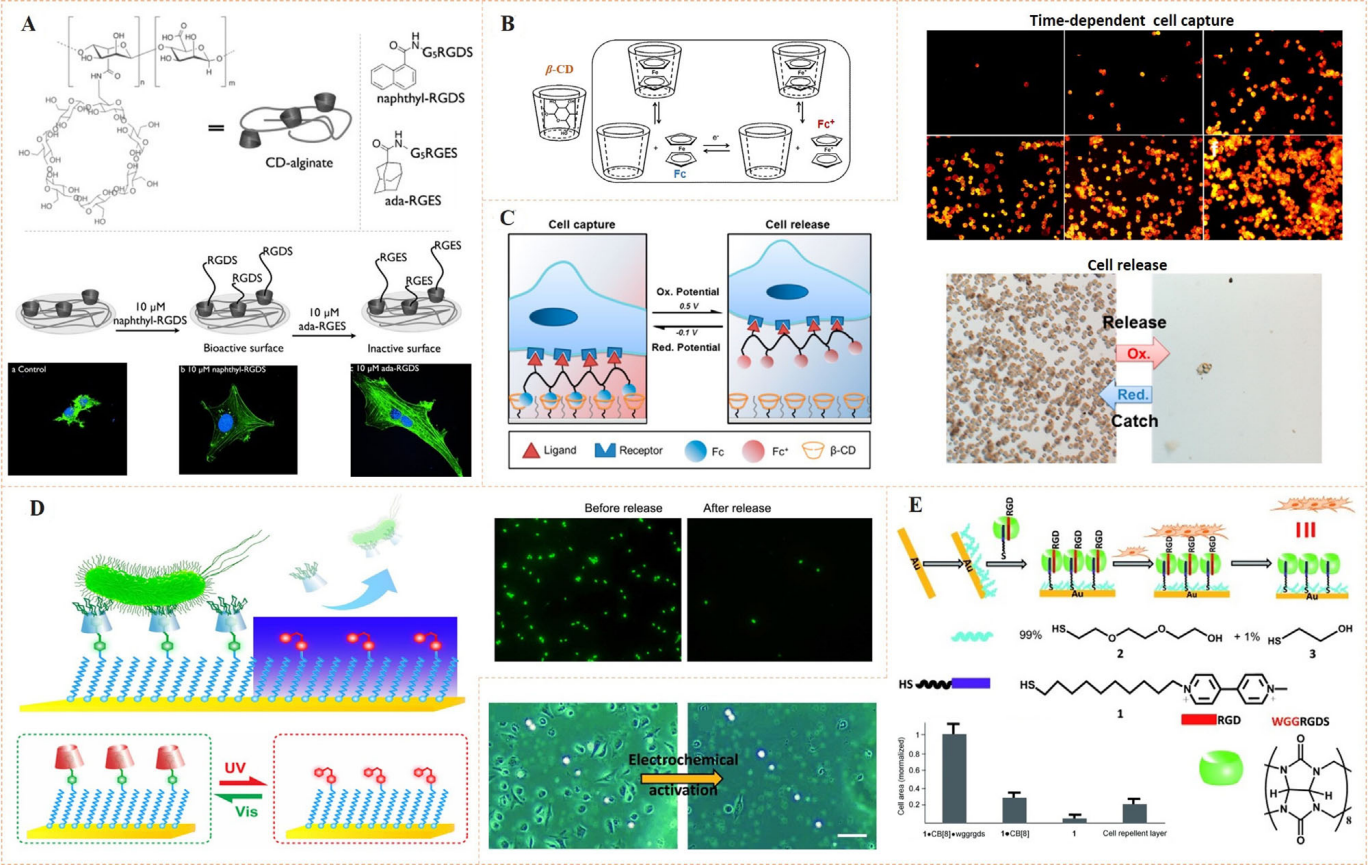
Host–guest supramolecular strategy for dynamic bioligand presentation. (A) A typical host–guest supramolecular dynamic biointerface using reversible adamantane and β‐CD interactions for controlled cell adhesion. Reproduced with permission.^[^ ^90^ ^]^ Copyright 2013, John Wiley and Sons. (B) The redox responsiveness of an Fc‐β‐CD host–guest complex. Reproduced with permission.^[^ ^94^ ^]^ Copyright 2014, Royal Society of Chemistry. (C) Schematic illustration shows the potential‐responsive system based on Fc‐β‐CD interactions for cell capture and release. Efficient and reversible cancer cell capture/release was shown in the microscope photos. Reproduced with permission.^[^ ^92a^ ^]^ Copyright 2017, American Chemical Society. (D) Schematic illustration of multivalent azobenzene‐β‐CD supramolecular platform for bacteria capture and release. Reproduced with permission.^[^ ^93a^ ^]^ Copyright 2017, American Chemical Society. (E) The strategy to dynamically introduce WGG‐terminated RGD peptide on Au SAM layer through CB[8]‐based host–guest interactions. The dynamic surface enabled electrical potential‐responsive RGD release and cell detachment. Reproduced with permission.^[^ ^14^ ^]^ Copyright 2012, John Wiley and Sons

Stupp and co‐workers first employed the CD‐based host–guest chemistry for dynamic display of cell adhesive ligand RGD (Figure 12A).^[^ ^90^ ^]^ They functionalized a β‐CD‐containing surface for binding of different highly hydrophobic guests such as adamantane (ada) and naphthyl modified with the peptide ligands. The dynamic binding was demonstrated through a molecular exchanging process between adamantane and naphthyl groups. Naphthyl possesses a weaker binding affinity toward β‐CD than adamantane, thus an inactive ada‐RGES is expected to displace the β‐CD‐bound naphthyl RGDS. This molecular exchange was confirmed by the result of cell adhesion, in which the average spreading area of adhered cells (with a typical focal adhesion) was greatly reduced after the addition of inactive ada‐RGES. This significant cell response demonstrated that the bioligands could be well controlled in a dynamic manner by host–guest chemistry.

The host–guest chemistry based on CD has been extensively studied in the past few years, and it has now been typically utilized for dynamic binding of different bioligands^[^ ^91^ ^]^ and reversible recognition of not only mammalian cells^[^ ^14^, ^24^, ^92^ ^]^ but also pathogenic bacteria.^[^ ^16^, ^93^ ^]^ Thereinto, novel guest molecules capable of introduction of photo‐ or electric responsiveness may bring new opportunities, for example, the spatiotemporal manipulation of cell–material interactions. Recently, Gao et al. designed an electrochemical chip based on host molecule β‐CD and guest molecule ferrocene (Fc).^[^ ^92a^ ^]^ The hydrophobic organometallic compound Fc can be from Fc‐β‐CD complexes in both organic and aqueous solutions. By electrochemical means, the Fc moiety can be oxidized to ferrocenium cation (Fc^+^), leading to a decrease of the complex formation constant (Figure 12B).^[^ ^94^ ^]^ In Gao's work, folic acid (FA), a cancer‐targeting bioligand, was co‐modified with Fc on a polyetherimide polymer (FA‐PEI‐Fc). It was found that FA‐PEI‐Fc could be immobilized on β‐CD‐functionalized chip in the reduced state (−0.1 V). In this case, cells can be captured on the chip within 15 min. In the oxidation state (0.5 V), Fc could be electrochemically oxidized into Fc^+^ and disassociated from the chip as a result of release of the captured cells (95% within 25 min) (Figure 12C). Moreover, cell density and pulse current demonstrated a linear association in the range of 100–1000 cells/cm^2^. This work shows the promise of electrochemical host–guest strategy in cell‐based cancer diagnosis.

Similar to the mechanism of electroactive guest‐caused electrochemical disassociation, photo‐sensitive guest molecules can also be used to from complexes with photocleavability. For instance, in the azobenzene (Azo)‐β‐CD host–guest complex, the β‐CD host has a better thermodynamic preference for *trans*‐Azo over *cis*‐Azo, indicating photoresponsive Azo guest binding and release. In view of this, Zhan et al. designed an OEG‐based photoresponsive SAM on gold substrate terminated with mixed Azo moiety (Figure 12D).^[^ ^93a^ ^]^ Meanwhile, β‐CD molecule was decorated with multiply mannose (CD‐M) and used as the bioligand‐modified host part. Due to the photoisomerization of Azo group, the Azo‐containing SAM showed efficient binding of CD‐M under dark while a rapid release of them under 365 nm of UV exposure. In the CD‐M binding state, the system enabled high concentration and specificity for the mannose‐specific type 1‐fimbriated bacteria capture. After being exposed with UV light (365 nm), the captured bacteria cells exhibited substantial detachment from the surface in 30 min. This work indicated the diversity and versatility of host–guest supramolecular chemistry for dynamic biomodification and modulation cell–biomaterial interactions. Besides the great potential for fabrication of smart diagnostic devices, this work also implied that a combination of dynamic covalent azobenzene with noncovalent host–guest chemistry may bring more possibilities in developing diverse functions, which are probably hard to be realized using single dynamic molecular strategy.

In addition to CD hosts, cucurbit[*n*]uril (CB[n]) is another macrocyclic host containing *n* glycoluril units.^[^ ^95^ ^]^ They have hydrophobic cavity and polar carbonyl around the portals, thus being capable of formation of stable host–guest complexes with charged hydrophobic molecules like alkyl ammonium ions, protonated adamantanamines, and so on.^[^ ^96^ ^]^ The methylviologen dications, such as *N,N’*‐dimethyl‐4,4′‐bipyridinium, can form a hetero‐guest pair with other molecules (e.g., aromatic groups) and bind with CB[8] resulting in a ternary host–guest complex.^[^ ^97^ ^]^ Jonkheijm's group first employed this ternary host–guest chemistry and demonstrated its potential for dynamic bioligand presentation (Figure 12E).^[^ ^14^ ^]^ In this work, an RGDS‐containing peptide with a WGG (Trp‐Gly‐Gly) sequence at the N‐terminal was synthesized and a viologen‐functionalized SAM on gold surface was fabricated. The WGG sequence could pair with viologen and form a ternary compound that is stable with CB[8].^[^ ^97a^ ^]^ The authors verified that the formation of CB[8]·viologen·WGGRGDS ternary complex on SAM could significantly improve surface cell adhesion. When a potential of −0.5 V was applied, the viologen groups were reduced, leading to a release of WGGRGDS. This dynamic behavior was verified by surface plasmon resonance (SPR) spectroscopy with in situ cyclic voltammetry. In the cell adhesion experiments, the adherent cells exhibited a significant detachment (over 90%) by simple washing, upon the application of an electrical potential (−0.5 V). The work demonstrated that CB‐based host–guest chemistry is another feasible strategy for dynamic bioligand presentation.

#### 2.2.2 DNA aptamer base pairing

DNA pairing is one of the ubiquitous molecular recognition events in biosystems. The base pairs of two DNA single strands can complementarily bind together through multiple noncovalent bonds (e.g., hydrogen bond, electrostatic force, and hydrophobic interactions). DNA aptamers isolated from the pool of random‐sequence libraries for bioligand binding are typically fabricated DNA or RNA molecules with a single strand. DNA aptamer complexes possess unique characterizations like hybridization,^[^ ^20^, ^98^ ^]^ isomerization,^[^ ^15b^ ^]^ complementation,^[^ ^99^ ^]^ and responsiveness (e.g., enzyme, pH, and temperature).^[^ ^15^, ^21^ ^]^ They can be used to bind to metal ions, drugs, proteins, and even receptors on the cell membrane.^[^ ^100^ ^]^ In view of this, researchers have employed DNA complementary sequences on the biomaterial interfaces for reversible capture and release of bioligands.

**FIGURE 13:**
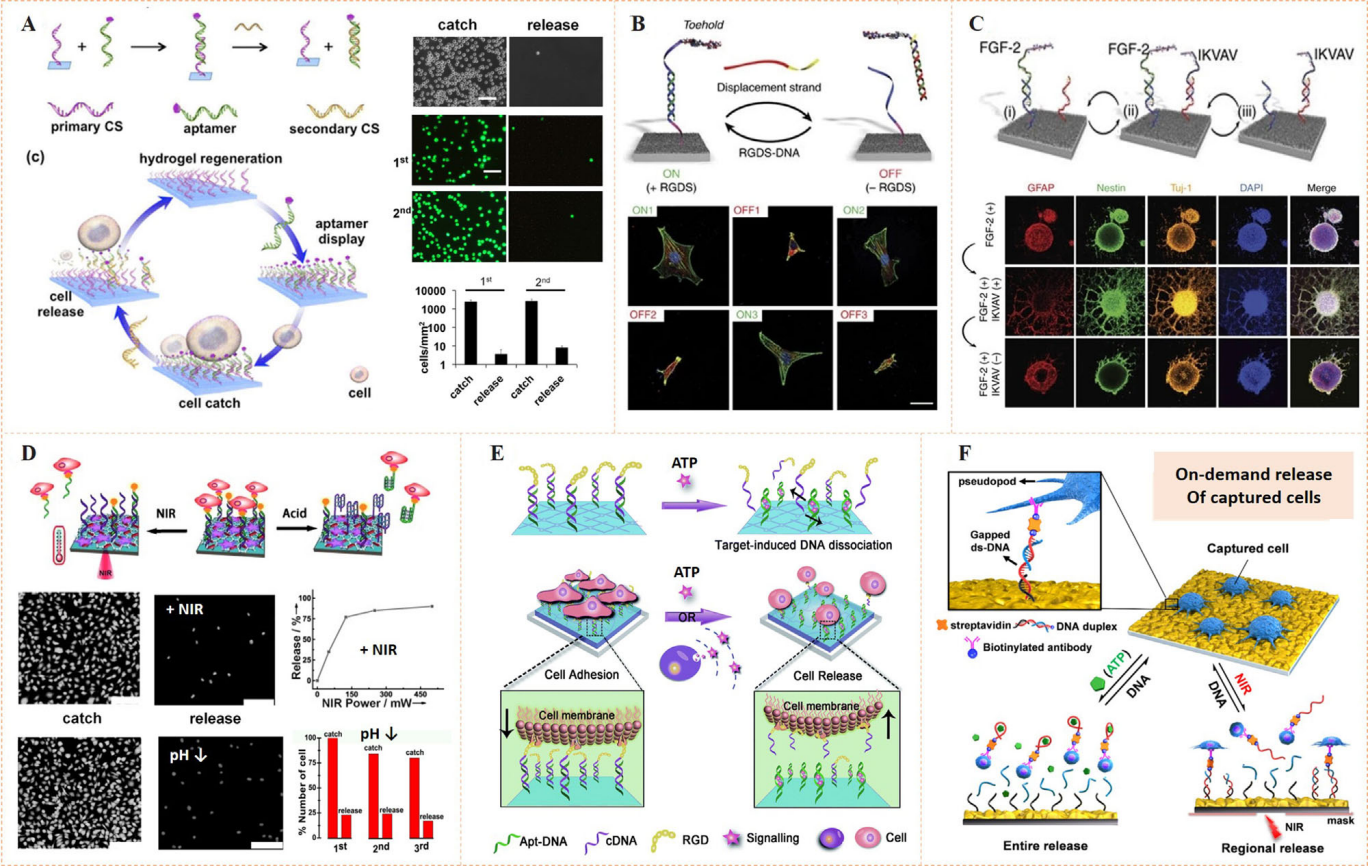
Dynamic bioligand presentation through DNA base pairing. (A) The preparation of a programmable hydrogel. Cell capture and release through the transformation of the aptamer with cell‐binding terminal sequence. Reproduced with permission.^[^ ^98^ ^]^ Copyright 2012, American Chemical Society. (B) Schematic of toehold‐mediated strand displacement system with reversible switching of cell adhesion. (C) Immunofluorescence images of neurospheres exposed to IKVAV and FGF‐2 signals in a dynamic and orthogonal presentation. Reproduced with permission.^[^ ^101^ ^]^ Copyright 2017, Nature Publishing Group. (D) The NIR‐ and pH‐responsive mechanism for dynamic cell binding. Microscopy images indicated the NIR and pH dual responsiveness for cell capture and release. Reproduced with permission.^[^ ^102^ ^]^ Copyright 2013, John Wiley and Sons. (E) Dynamic regulation of cell adhesion by using ATP‐responsive DNA aptamer‐based hydrogel. Reproduced with permission.^[^ ^103^ ^]^ Copyright 2015, Royal Society of Chemistry. (F) Schematic illustration of DAN‐based surfaces with ATP‐ and NIR‐responsive control of cell recognition and release. Reproduced with permission.^[^ ^99^ ^]^ Copyright 2018, American Chemical Society

Zhang et al. engineered a DNA aptamer‐bearing polyacrylamide hydrogel surface with the property of cell capture and release through DNA competitive hybridization (i.e., strand displacement mechanism) (Figure 13A).^[^ ^98^ ^]^ The surface single stranded aptamers were further hybridized with cell‐specific aptamer‐linked complementary sequences, leading to a cell‐targeting surface. With these cell‐specific aptamers, the surface could bind and capture target cells specifically. Upon the addition of a secondary complementary DNA chain, the initial hybridized cell‐specific sequence was competed and replaced, leading to the dissociation of cell‐specific aptamers from the hydrogel. In conclusion, the capture cells were rapidly released from the hydrogel along with the dissociated cell‐specific aptamers (about 95% of the cells could be released in 10 min). Since cell capture/release process was triggered by aptamer hybridizations, the system showed high biocompatibility, reproducibility, and programmable cell regulation.

DNA competitive hybridization can also be used to control multiple bioligands orthogonally and reversibly. The idea was well demonstrated by Stupp and co‐workers (Figure 13B,C).^[^ ^101^ ^]^ In their work, dynamic cell adhesion was first demonstrated using RGDS‐capped DNA on surface with complementary DNA sequences. The DNA competitive hybridization could lead to switchable RGDS presentation (ON/OFF). Thus, reversible cell adhesion was observed (Figure 13B). In addition, two epitope peptides IKVAV and FGF‐2 peptide, from laminin‐1 and mitotic growth factor fibroblast growth factor 2, were employed for the differentiation and proliferation of neural stem cells. Corresponding to the toehold sequences, two orthogonal peptide‐DNA (IKVAV‐ or FGF‐2‐DNA) complexes were synthesized. The two peptide DNAs could be surface immobilized by the complementary DNA hybridization. The surface bioactivities derived by peptide could be reversible “on” and “off” by adding complementary DNA trigger. Moreover, the bioactive peptides could be displayed in two different dynamic ways. The inclusion of the peptide‐DNA in a programmed manner might result in the successive presentation of the bioligands throughout time; and simultaneously displaying both peptides, the detachment of each peptide at different time points could also be performed independently and selectively (Figure 13C). Such a kind of sequential bioligand presentation may enable the controllable proliferate of stem cells first, and following that selective differentiation by activation of another bioligand. This dynamic biointerface is very attractive for developing the dynamic cell niche in tissue engineering and regenerative medicine. Fan's group also employed the programmable mechanism of strand displacement reactions to instruct cell adhesion by DNA‐based on‐chip logic gates.^[^ ^104^ ^]^ Due to the sequential recognition and strand displacement mechanism, cell adhesion can be logically regulated as a result of a Boolean combination of ssDNA inputs. This work showed that the DNA‐based computing components for biological logic gates could provide the tool that is highly generic for programmable regulation of cell behaviors with high precision and predictability.

In addition to strand displacement, DNA aptamers can undergo conformational changes (i.e., isomerization) in the presence of target molecules, ions, or photo‐thermal stimulation. For example, K^+^ can induce the AS1411 aptamer to form G‐quadruplex structure, which has high affinity to tumor cell membrane nucleolin;^[^ ^105^ ^]^ adenosine triphosphate (ATP) could bind with ATP‐DNA aptamers, leading to a stable tertiary structure.^[^ ^106^ ^]^ These target‐triggered isomerizations of DNA aptamers can also use dynamic display bioligands. Qu's group has developed several dynamic substrates with molecule, ion, or photoresponsiveness for dynamic control of cell adhesion.^[^ ^102^, ^103^ ^]^ The authors demonstrated that pH‐ and NIR‐responsive dynamic substrates could be prepared using thermosensitive C‐rich DNA. The pH‐ or NIR‐controlled assembly and disassembly of duplex DNA resulted in the binding and release of the complementary DNA modified with RGD ligand, showing a reversible cell catch‐and‐release on the biointerface (Figure 13D).^[^ ^102^ ^]^ In a further work, the same group turned their attention to the biological molecule responsiveness in the design DNA aptamer‐based dynamic systems (Figure 13E).^[^ ^103^ ^]^ They employed ATP‐DNA aptamer to introduce ATP responsiveness, because the ATP is an important exogenous molecule closely associated with both physiological and pathological mechanisms. After grafting on a cell‐repellant alginate hydrogel surface, the ATP aptamer could hybridize with complementary DNA modified by RGD (RGD‐DNA). Compared with the bare surface, the surface presentation of bioactive RGD exhibited better cell adhesion. Upon the addition of ATP molecules, the ATP‐DNA aptamers, however, underwent a rapid conformational transition, resulting in RGD‐DNA dissociation and release. At the same time, the RGD‐mediated cell adhesion was weakened, and cell detachment occurred. Gao et al. further developed DNA‐based dynamic surface with NIR and ATP dual responsiveness for cancer call capture and release (Figure 13F).^[^ ^99^ ^]^These studies suggested that DNA base paring can be well designed in response to exogenous biosignals, and this would greatly enhance the self‐controllability for manipulation of cellular functions according to biofeedback mechanisms.

#### 2.2.3 Tunable intermolecular interaction

The dynamics of ECM are commonly reflected in processes of natural molecular recognition events, which mainly rely on reversible intermolecular bindings in receptor–ligand, antibody–antigen, DNA–protein, RNA–ribosome, sugar–lectin, and so on.^[^ ^107^ ^]^ These intermolecular interactions are commonly noncovalent, for example, hydrogen bonds, coordination, hydrophobic forces, π–π interactions, van der Waals forces, electrostatic effects, and so on. Synthetic polymers with specific functional groups also possess these reversible bindings toward biomolecules (i.e., chemically designed molecular recognition).^[^ ^108^ ^]^ Over the past decade, stimuli‐responsive polymers have raised more interests in researchers due to their adjustable physicochemical features for altering intermolecular interactions. Typically, stimuli‐responsive polymers are a class of synthetic macromolecules that may alter conformation or structure based on changes in light, temperature, pH, electric or magnetic field.^[^ ^109^ ^]^ These external stimuli can cause dramatic changes in the polymer properties like charge state, hydrophilicity/phobicity, conductivity, mechanical and other physical properties. Since the charge state and wettability are critical for reversible intermolecular bindings,^[^ ^110^ ^]^ these stimuli‐responsive polymers thus have great potential for reversible and controlled bioligand binding at a biomaterial interface.

**FIGURE 14:**
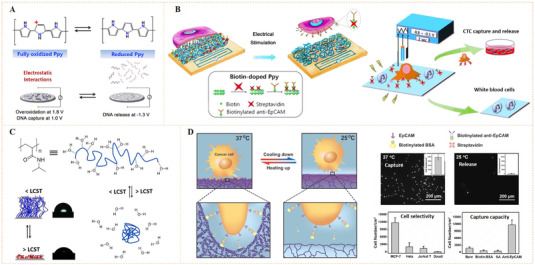
Reversible binding of bioligand through tunable intermolecular interactions. (A) Schematic illustration of the electric field‐mediated redox of Ppy for reversible biomolecular binding. Reproduced with permission.^[^ ^114c^ ^]^ Copyright 2016, Elsevier B.V. (B) Anti‐EpCAM‐immobilized on the biotin‐doped Ppy surface with “on‐demand” release of captured cells through the application of a direct current electric field. Reproduced with permission.^[^ ^115^ ^]^ Copyright 2014, John Wiley and Sons. (C) The thermo‐responsive property of PNIPAAm. (D) Scheme illustration of thermo‐responsive hydrogel surface with hydrophobically bound antibodies for cancer capture (37°C) and release (25°C). Reproduced with permission.^[^ ^116^ ^]^ Copyright 2014, John Wiley and Sons

Conductive polymers, for example, polypyrrole (Ppy)^[^ ^111^ ^]^ and polythiophene (PTH)^[^ ^112^ ^]^ can occur reversible redox reaction under electric field, leading to changes in charge. They have been commonly applied in dynamic surfaces for reversible capture and release of specific biomolecules such as DNA, protein, cells, bacteria, and so on.^[^ ^113^ ^]^ Ppy is one of the most commonly used conductive polymers as drug carriers or medical implants since it can electrostatically bind with a variety of peptides, proteins, and DNAs.^[^ ^114^ ^]^ Moreover, the bound biomolecules on a Ppy surface can be specifically released when applied with electric fields (Figure 14A).^[^ ^114c^ ^]^ Jeon et al. produced a novel biotin‐capsulated Ppy substrate for selectively capturing and releasing cancer cells (Figure 14B).^[^ ^115^ ^]^ The biotin molecules were bound directly onto Ppy substrate during pyrrole electrochemical polymerization. The densely packed biotin could bind with streptavidin‐tagged anti‐EpCAM antibody, leading to a bioactive surface that enabled the recognition of EpCAM‐positive cancer cells. When the cell‐bound surface was applied with a weak electrical stimulation, the reduction of Ppy occurred and resulted in a reduced Ppy with no surface charge. This electrochemically induced shift in the surface charge state result in the release of the biotin and the bioligands linked to it. The authors demonstrated that the anti‐EpCAM‐biotin‐Ppy platform was capable of selective capture of cancer cells from blood and efficient release of them following the application of a nondestructive negative potential.

Besides electro‐responsive conductive polymers, thermo‐responsive polymers represent another type of functional molecules capable of reversible interaction with various biomacromolecules.^[^ ^117^ ^]^ Typically, thermo‐responsive polymers are known for its reversible conformational transition in aqueous solution induced by temperature.^[^ ^18^, ^118^ ^]^ Poly(*N*‐isopropylacrylamide) (PNIPAAm) is the most extensively investigated chemical among thermo‐responsive polymers. PNIPAAm is able to undergo a transition from expanded coil to compact globule in aqueous solutions in the case of temperature above the lower critical solution temperature (LCST, ∼32^o^C). The conformational transition of PNIPAAm in a surface coating format commonly showed dramatic changes in the surface wettability (Figure 14C). Due to the reversible hydrophilic/phobic conversion, PNIPAAm‐grafted surfaces have been widely used to control the dynamic binding of biomolecules.^[^ ^119^ ^]^ Hydrophobic interactions represent one of the universal noncovalent interactions in biological events including the phospholipid bilayer formation, protein folding, receptor–ligand, and antibody–antigen recognitions. In view of this, Wang's groups first employed the surface wettability of PNIPAAm for bioligand binding and control of reversible cell capture and release.^[^ ^116^, ^120^ ^]^ In a recent work, they prepared a thermo‐responsive PNIPAAm‐based thin hydrogel layer. A biotinylated bovine serum albumin (biotin‐BSA) was bound on the hydrogel through strong hydrophobic interactions between BSA and the PNIPAAm at 37°C (Figure 14D). Subsequently, the cancer cell‐targeting agent, biotinylated anti‐EpCAM, was bound on the biotin–BSA surface through biotin–streptavidin recognitions. The anti‐EpCAM‐bound surface could selectively capture human breast cancer (MCF‐7) cells at 37°C (above LCST). Subsequently, the temperature was decreased to 25°C (below LCST), a change of hydrogel surface wettability from hydrophobicity to hydrophilicity occurred. As a result, the hydrophobic driving force between biotin and PNIPAAm hydrogel vanished, allowing the trapped cells to be released rapidly. In another work, the author also combined topographic effects with thermo‐responsive hydrophobic interactions for the capture/release of cancer cells.^[^ ^120^ ^]^ These studies jointly demonstrated that PNIPAAM‐mediated hydrophobic interaction is a simple and efficient noncovalent strategy for dynamic bioligand presentation.

#### 2.2.4 Molecularly imprinted biorecognition

**FIGURE 15:**
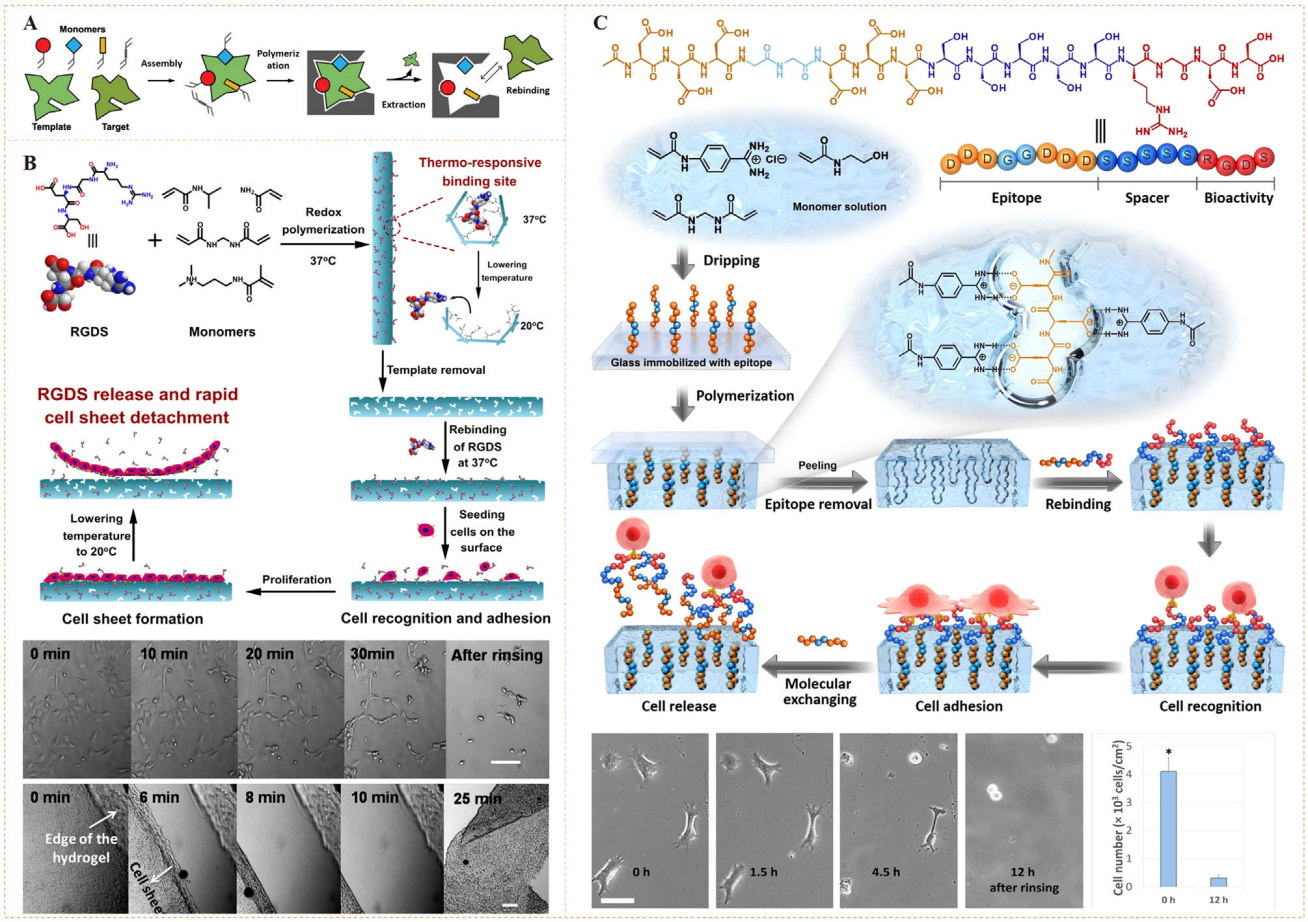
Dynamic bioligand presentation through molecular imprinting. (A) The principle of molecular imprinting technology. Reproduced with permission.^[^ ^122^ ^]^ Copyright 2011, Biochemical Society. (B) Thermo‐responsive RGD‐binding hydrogel surface for reversible cell adhesion and cell sheet harvesting. Reproduced with permission.^[^ ^123^ ^]^ Copyright 2013, John Wiley and Sons. (C) Schematic illustration of the epitope‐imprinted hydrogel surface for dynamic display of RGD peptide and controlled cell adhesion through molecular exchange. Reproduced with permission.^[^ ^124^ ^]^ Copyright 2017, John Wiley and Sons

Molecular imprinting (MI) belongs to the category of supramolecular chemistry capable of generating specific recognition sites on a polymeric substrate toward the template molecules. The mechanism of MI is similar to the “lock and key” of natural receptor–ligand recognitions, thus molecularly imprinted polymers (MIPs) are also well recognized as synthetic receptors or plastic antibodies.^[^ ^121^ ^]^ Molecular imprinting process typically involves three steps: molecular pre‐assembly, polymerization, and template removal (Figure 15A).^[^ ^122^ ^]^ In consequence, imprinted sites with comparable shape, size, and functionality complementary to the template molecules can be created. Commonly, MIPs rely on noncovalent interactions to achieve molecular recognition. Thus, the intrinsic reversibility of MI‐mediated molecular recognition would be a potential dynamic driving force, just like the host–guest recognitions, for the dynamic bioligand presentation. Moreover, given the restricted chemical techniques for achieving dynamic ligand presentation, such a molecularly adjustable dynamic system may bring more opportunities to this field.

Theoretically, the MI process can be employed to tailor specific recognition toward diversified molecules (e.g., small organic molecules, peptides, and even large proteins).^[^ ^121^, ^125^ ^]^ Fukazawa and Rosellini first attempted to use fibronectin (FN)‐imprinted surface to study the cell adhesion or proliferation behaviors.^[^ ^126^ ^]^ Likewise, Wu et al. prepared an ovalbumin‐imprinted cell adhesive membrane.^[^ ^127^ ^]^ However, the above studies cannot obtain dynamic biomolecular binding and reversible cell behaviors, probably due to the weak affinity and poor reversibility of the MIPs. Given this, Pan and co‐workers prepared a PNIPAAm‐based, RGD peptide‐imprinted hydrogel layer for regulating reversible cell adhesion. The imprinted hydrogel showed specific affinity toward the cell adhesive peptide RGD, and the thermoresponsiveness endowed the imprinted hydrogel with tunable RGD affinity (Figure 15B).^[^ ^123^ ^]^ At the physiological temperature (i.e., 37°C, above the LCST of PNIPAAm), the hydrogel could stably bind with RGD, thus showing good cell adhesion behavior. Interestingly, when the system was cooled down to 20°C (blow the LCST), adhered cells could be detached in a short time, because of the release of temperature‐triggered RGD peptides and change of surface wettability. This work, in a first attempt, verified the applicability of MI for dynamic bioligand presentation and reversible modulation of cell–material interactions. Moreover, a physiologically adaptable temperature range (20–37°C) was employed in this work, indicating that the reversible cell activities could be managed noninvasively.

To improve the accessibility of an imprinted bioligand, Pan and co‐workers further create an epitope imprinting technique for dynamic bioligand illustrate and cell adhesion control. (Figure 15C).^[^ ^124^ ^]^ Typically, an epitope imprinting method refers to the utilization of a terminal peptide sequence in a long peptide or protein as the template for imprinting, and the resultant epitope‐imprinted sites can recognize the whole long peptide or protein by means of specific binding toward the epitope part. In this work, the authors fabricate an epitope peptide‐imprinted hydrogel surface using microcontact method. The epitope‐imprinted surface thus enabled to recognize an RGD‐capped long peptide and left the terminal RGD ligand to free up for cell recognition. Binding assays demonstrated that the epitope‐imprinted surface could not only stably bind RGD‐capped long peptide but also detach it through a template epitope‐triggered molecular exchange. With RGD bound on the surface, cells could specifically adhere. Upon a sufficient quantity of the free epitope peptide was added to the medium, a steady transformation in cell morphology from spreading to round was observed, indicating dynamic changes of cell adhesion behaviors. This work confirmed the viability of MI for the construction of synthetic biointerface with dynamic ligand presentation one more time. More excitingly, cell behaviors were controlled by a particular biomolecule that could be tailored throughout the imprinting process. Furthermore, we can also imagine that, by using stimuli‐responsive polymer networks, the molecularly imprinted biorecognition can be equipped with more diverse control means, facilitating the manipulation of more complex cell behaviors.

#### 2.2.5 Library‐screened biorecognition

**FIGURE 16:**
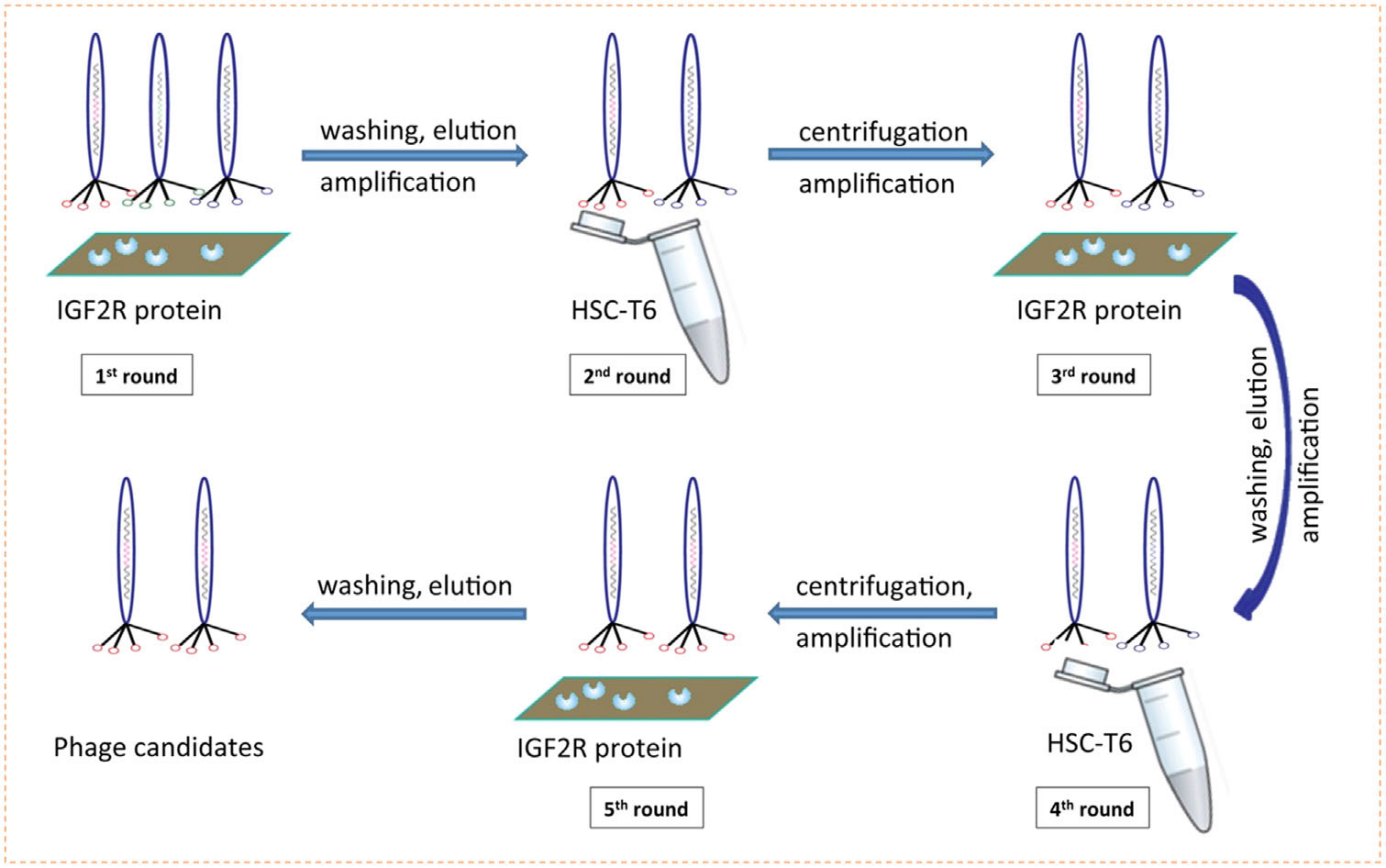
Overview of the phage display selection process to screen high affinity biomolecules (e.g., peptides, or proteins). Reproduced with permission.^[^ ^130^ ^]^ Copyright 2015, American Chemical Society

The success of MI for surface bioligand presentation provides a hint that the artificially designed molecular recognition is an emerging class of dynamic driving forces for biomolecular conjugations. Apart from the molecularly imprinted templating method, the library‐based affinity selection technology can also generate specific molecular recognition by mimicking the natural evolution through screening and identifying ligand–target interactions among thousands of potential targets.^[^ ^128^ ^]^ Schrader's group first attempted to screen and identify protein‐affinity polymers by using a random statistical linear copolymers library.^[^ ^128b^ ^]^ With a similar procedure, Shea and co‐workers employed this concept for the discovery of cross‐linked polymeric nanoparticles with peptide or protein affinity.^[^ ^128c^ ^]^ After screening from a library of synthetic polymer nanoparticles or linear copolymer incorporating various functional monomers yields receptor‐like candidates with excellent affinity and selectivity to targeted biomacromolecules. However, the chemical library based on synthetic molecules has limited variants as compared to the vast number of natural ligands. In contrast, the phage display libraries contain up to 10^10^ variants, which can be constructed simultaneously.^[^ ^129^ ^]^ Phage display technology is another well‐established functional genomics method based on the use of filamentous phages,^[^ ^128g^ ^]^ which can obtain peptides or proteins with high affinity and specificity toward numerous types of targets (Figure 16).^[^ ^130^ ^]^ Phage display has shown great success in many areas including immunology, cell biology, pharmacology, and drug discovery.

To date, plenty of peptides have been identified with specific affinity toward cell membrane receptors or growth factors through the phage display technology.^[^ ^130^, ^131^ ^]^ Similar to the MI‐mediated molecular recognition, the phage display library‐screened peptide–protein affinity is also intrinsically reversible. This implies the possibility to fabricate peptide‐functionalized biomaterials for dynamic bioligand presentation. Stupp's group first used this strategy to modify a peptide‐assembled nanofiber surface.^[^ ^131b^ ^]^ They discovered a short peptide (HSNGLPL) by phage display, which shows specific binding affinity to the growth factor TGF‐β1. By linking a peptide amphiphile to the end of the HSNGLPL peptide, a nanofiber‐based dynamic nanointerface with reversible TGF‐β1 presentation was readily obtained. Due to the intrinsic affinity of HSNGLPL peptide, the nanofiber biomaterials could bind and release TGF‐β1 in vitro. More importantly, the material could recruit endogenous TGF‐β1 in vivo. This process is reminiscent of the binding and releasing of growth factors to components of the ECM (such as collagens and heparin) that occurred in biological systems. The authors also confirmed that the dynamic biomaterials could promote the repair of defected cartilage tissue in a rabbit model without the addition of exogenous TGF‐β1. The study demonstrates not only the possibility of library‐screened biorecognition for dynamic bioligand presentation, but also the potential of these dynamic biomaterials in tissue engineering and regenerative medicine.

## 3 BIOLOGICAL EFFECTS AND APPLICATIONS

As a relatively simple and conceptual template of ECM, dynamic biointerface with tunable functional bioactivity represents an essential quality indispensable to the next generation of biomaterials. Although these synthetic dynamic biointerfaces are far from the ingenuity of a natural ECM, they have still exhibited significance in biomedical science from foundational research to applications. We have discussed these dynamic biointerfaces on the basis of the dynamic molecular strategies. Some of their biological effects or potential applications have been roughly mentioned. In this section, we separately highlighted the uses of these ECM‐mimicking dynamic surfaces, and emphasis was put on some typical examples with generality or significance.

### 3.1 Cell adhesion regulation

As one of the basic cell behaviors, reversible cell adhesion is involved in almost all kinds of cellular processes. For instance, at the damage sites, successful tissue self‐repair is dependent on a continuous and reciprocal conversion between cell attachment and detachment;^[^ ^132^ ^]^ and cancer metastasis similarly involved a tumor cell detachment from the solid tumor and their re‐assembly along the blood artery.^[^ ^133^ ^]^ Therefore, it is necessary to study this ubiquitous dynamic on an artificial platform, which may be helpful for the exploration of pathological and physiological mechanisms.

As discussed above, dynamic molecular strategies have been extensively explored and cell adhesion could be well manipulated by dynamic bioligand presentation.^[^ ^7h^ ^]^ RGD‐mediated integrin‐targeting represents the most extensively studied bioligand model. To date, dynamic display of RGD on biomaterial interfaces has been well demonstrated with dynamic cell adhesion through external stimuli like temperature, NIR/UV light, magnet, electric potential, pH, organic molecule, sugar, GSH, peptide, enzyme, DNA, etc. However, most of the systems cannot exhibit complete reversibility in RGD presentation, especially for these with dynamic mechanisms based on ligand release but are unable to recycle them in an easy‐to‐handle manner. Azobenzene photoisomerization represents the most essentially reversible and easy‐to‐handle molecular strategy for ligand presentation.^[^ ^134^ ^]^ The activated or inactivated status can be readily and completely reversed by alternating the exposure under UV light. However, the cytotoxicity of azobenzene and potential invasiveness of UV light to biosystems compromise its practical applications.

**FIGURE 17:**
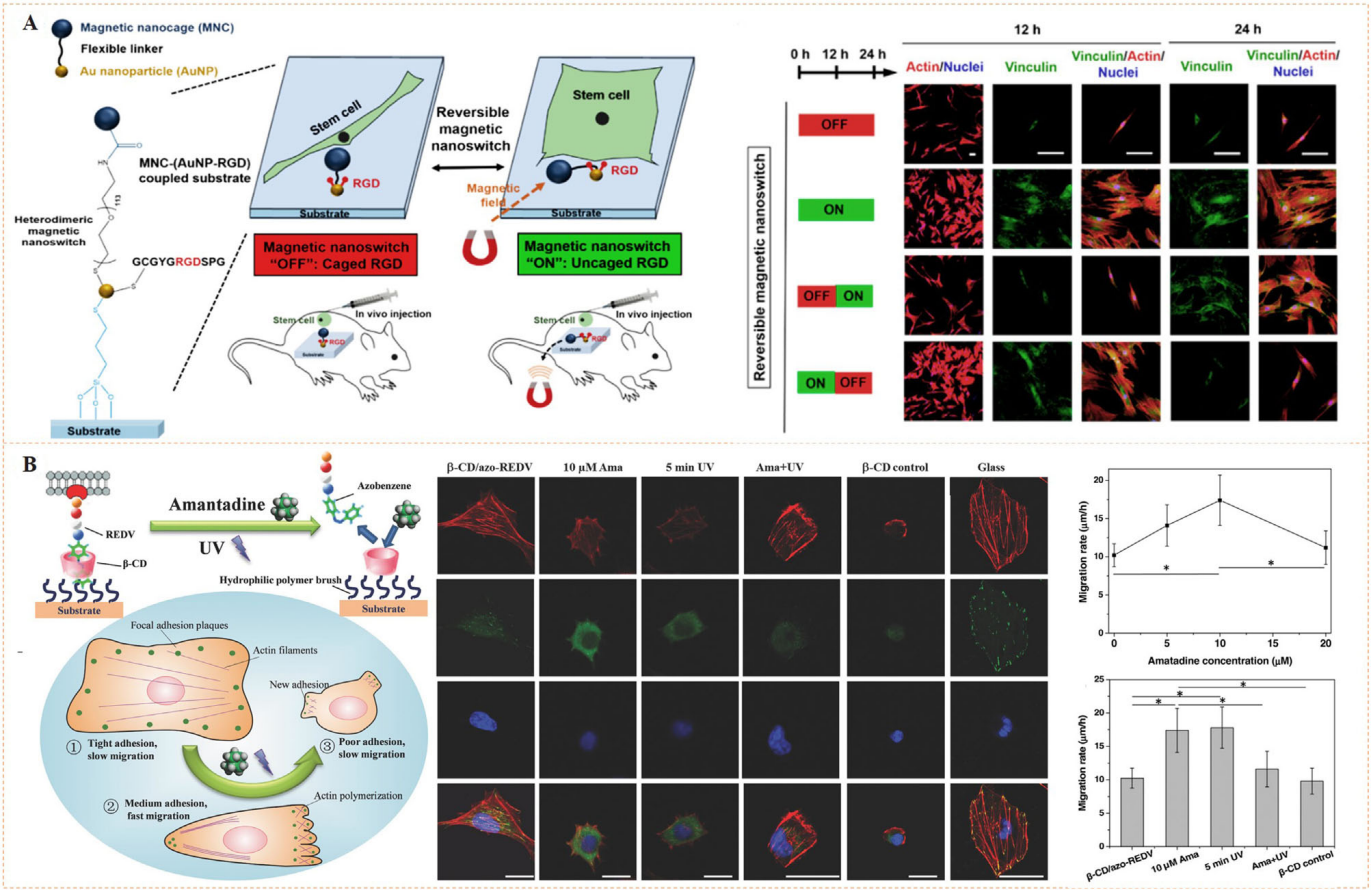
(A) Schematic illustration (left) of the heterodimeric magnetic nanoswitch for in vitro and in vivo regulation of adhesion of stem cells by reversibly manipulating RGD ligand caging and uncaging. Time‐dependent immunofluorescent staining images (right) of cells on the substrate by reversibly turning on and off the RGD ligands. Scale bars represent 50 μm. Reproduced with permission.^[^ ^135^ ^]^ Copyright 2018, American Chemical Society. (B) Schematic (left) of the dual responsiveness of β‐CD/azo‐GREDV‐modified surface and the mechanism for endothelial cell migration behavior regulation under UV light or Ama stimulation. Fluorescent images and quantitative analysis (right) indicated the differences of cell adhesion and migration on different stimulations. Reproduced with permission.^[^ ^136^ ^]^ Copyright 2016, John Wiley and Sons

Inspired by the reversibility and easy handling property of azobenzene isomerization, Bian and co‐workers developed a magnetic field‐controlled, isomerization‐like bioligand hiding and exposing strategy for reversible regulation of cell adhesion (Figure 17A).^[^ ^135^, ^137^ ^]^ The system was based on an RGD‐decorated AuNP (AuNP‐RGD) capped with magnetic nanoparticles (MNs) through a long flexible linker on a substrate. Applying a magnetic field is as easy as UV light to implement, which could change the positions of MNs to hide or expose the RGD peptides on AuNP, thus obtaining a magnet‐responsive biointerface capable of remote control of bioligand presentation. This convenience thus facilitates the regulation of cell adhesion both in vitro and in vivo. The authors verified that the magnetic field‐controlled reversible bioligand functions could not only elicit dynamic focal adhesion and spreading, but also impact the mechanosensing and even differentiation of stem cells. This work thus provides a potential and feasible means capable of improving the outcomes of medical implants and enhancing the efficiency of tissue regenerative therapies involving stem cells. We anticipate that relevant studies using this platform for modulating in vivo stem cell differentiation will be realized in a near future.

Regarding reversible cell adhesion, we have to mention another cell behavior, that is, cell migration. In essence, the cell migration is a dynamic cell adhesion process involved in many biological events in development, wound repair, immune response, and metastasis. Regulation of cell migration thus is of importance to understand its fundamental functions and helpful for the design of biomaterials capable of directing cell movement. Previous studies have demonstrated that directional cell movement could be guided by free signal molecules, electric field, stiffness, and other physiochemical properties,^[^ ^138^ ^]^ while the effect of ligand–receptor interaction on cell migration has rarely been explored. Recently, Gao and co‐workers found that the density of bioligand was significant for cell migration, thus regulation of the bioligand presentation showed the possibility to control cell migration.^[^ ^138^, ^139^ ^]^ The same group developed a dual‐responsive surface based on molecular exchangeable β‐CD‐based host–guest interactions (Figure 17B).^[^ ^136^ ^]^ The β‐CD host can bind with adamantine (higher affinity), naphthalene (moderate affinity), *trans*‐azobenzene (low affinity) but not the *cis*‐azobenzene (negligible affinity). Due to variation of binding affinity, guest molecules with the bioligand (endothelial cells selective Arg‐Glu‐Asp‐Val, REDV) might be discharged in two regulated increments. The applications of different stimuli led to gradient change of the surface REDV ligand, thus showing the ability to dynamic mediate cell migration. This work, from a new perspective, demonstrated the significance of dynamic ligand presentation in tissue regenerative biomaterials.

### 3.2 Macrophage phenotype control

Macrophages, as a type of mononuclear phagocytes, are central to a variety of immune functions including host defense and maintenance of tissue homeostasis.^[^ ^140^ ^]^ Macrophages can polarize to different phenotypes, such as the classic pro‐inflammatory M1 and anti‐inflammatory M2 activation states, as well as regulatory phenotypes and other subtypes.^[^ ^141^ ^]^ Macrophages show important immune functions in tissue repair and regeneration. Upon tissue trauma, macrophage‐mediated immune responses activate. During this phase, macrophages (M1 phenotype) can clear cell debris, combat microbes, activate inflammation and promote fibrosis, and (M2 phenotype) activate stem/progenitor cells and remodel the ECM for regeneration to coordinate tissue healing processes. Previous studies have revealed that an efficient and timely switching of macrophage phenotype from pro‐inflammatory M1 to anti‐inflammatory M2 was essential during tissue healing.^[^ ^142^ ^]^ Thus, the control of macrophage phenotypes on the biomaterial interfaces is very essential, which is beneficial to balance the two sidedness of immune actions, for rapid tissue regeneration.^[^ ^143^ ^]^

**FIGURE 18:**
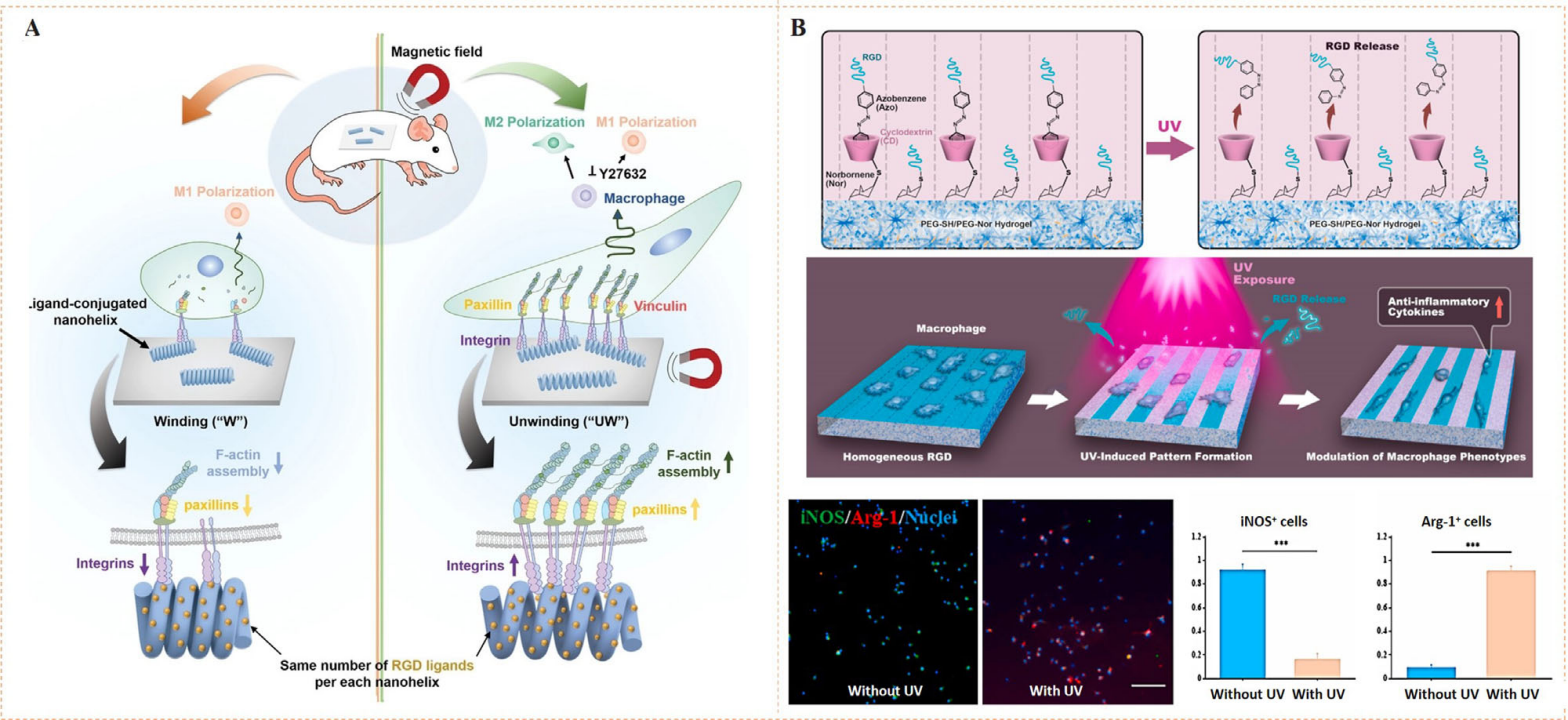
(A) Schematic illustration of surface magnetic manipulation through in situ winding (“W”) and unwinding (“UW”) of the helical ligand‐conjugated nanostructures for integrin recruitment to stimulate macrophage M2 polarization or inhibits integrin recruitment inhibition to induce macrophage M1 polarization, respectively. Reproduced with permission.^[^ ^144^ ^]^ Copyright 2021, John Wiley and Sons. (B) Schematic of the UV‐induced dynamic RGD pattern to sequentially modulate macrophage phenotypes between pro‐inflammation and anti‐inflammation. In the bottom are the fluorescence images and quantitative results of cells stained for iNOS (green), Arg‐1 (red), and nuclei (blue) on the dynamic RGD pattern with or without UV exposure. Reproduced with permission.^[^ ^145^ ^]^ Copyright 2021, Elsevier B.V

It has been found that the integrin‐targeting peptide RGD could regulate the adhesion and polarization of host macrophages. Different levels of RGD‐mediated integrin recruitment and adhesion assembly in macrophages result in distinct macrophage polarization directions.^[^ ^146^ ^]^ In this context, the group of Kang and Bian pioneered the uses of a series of dynamic RGD‐displayed surfaces for the regulation of macrophages.^[^ ^144^, ^147^ ^]^ RGD peptides in these studies could be dynamically controlled through reversible ligand‐cation coordination,^[^ ^147a^ ^]^ slidable nano‐ligand switch,^[^ ^147b^ ^]^ or winding/unwinding of nanohelical ligands,^[^ ^144^ ^]^ reversible nanoligand movement.^[^ ^147c^ ^]^ In essence, the mechanism of these dynamic systems was based on a ligand density variation strategy. For example, magnetic helical RGD‐conjugated nanostructures on the substrate that are capable of recurrent winding (“W”) and unwinding (“UW”) in nanoscale were used to regulate ligand density and manipulate macrophages (Figure 18A).^[^ ^144^ ^]^ Magnet‐induced in situ motion of the ligand‐conjugated nanohelices could increase or decrease ligand nanospacing, resulting in the stimulation of M2 or M1 polarization, respectively. The magnet‐responsive dynamic systems bring new ideas to introduce noninvasive and handy means for control of host responses to biomaterials.

In addition to magnet‐responsiveness, photoresponsive ligand presentation represents another promising means for high‐efficiency macrophage phenotype regulation. Very recently, Mao and co‐workers proposed a novel dynamic biointerface for spatiotemporal regulation of macrophage phenotype polarization using photo‐induced dynamic RGD pattern (Figure 18B).^[^ ^145^ ^]^ The system was based on the combined use of UV‐patterning technique and photoresponsiveness of β‐CD/azobenzene‐RGD (Azo‐RGD) complex. Polyethylene glycol‐dithiol/polyethylene glycol‐norbornene (PEG‐SH/PEG‐Nor) hydrogel modified with alternately patterned β‐CD was used as the substrate due to the cell repellent property. The formation of CD/Azo‐RGD complex led to a homogeneous RGD surface. Upon the irradiation under 365 nm UV light, the homogeneous RGD surface could be transformed to an RGD‐patterned surface, which could induce a round to elongated morphological change and also phenotypic transition from pro‐inflammation to anti‐inflammation of the adhered macrophages. This photoresponsive dynamic ligand‐patterned surface provides a remotely controlled strategy to regulate macrophage phenotypes, which can adapt the immune microenvironment to obtain an optimized tissue healing outcome in vivo.

### 3.3 Stem cell fate determination

As a type of “universal cells” that can differentiate into different cell phenotypes under specific conditions, stem cells are the fountainhead of organs or tissues.^[^ ^148^ ^]^ They have important significance in tissue engineering and regenerative medicine, and can be used for tissue repair or direct replacement of diseased tissues in clinical applications.^[^ ^149^ ^]^ The differentiation of stem cells can be stimulated by not only growth factors,^[^ ^150^ ^]^ the states of bioligand presentation are also closely associated with the fate of stem cells. For example, RGD‐induced focal adhesion formation and mechanosensing can mediate various cellular functions, especially stem cell differentiation.^[^ ^151^ ^]^ In this context, Bian's group explored the control of stem cell differentiation based on their previously developed RGD‐nanoligands.^[^ ^135^, ^137^ ^]^ They found that the ligand density variation through physical manipulation of the nanoligand movement could not only remotely regulate cell adhesion and macrophage phenotypes but also the differentiation of stem cells.^[^ ^81^, ^135^ ^]^ These studies unraveled that dynamic RGD presentation can stimulate mechanosensing‐mediated stem cell differentiation.

**FIGURE 19:**
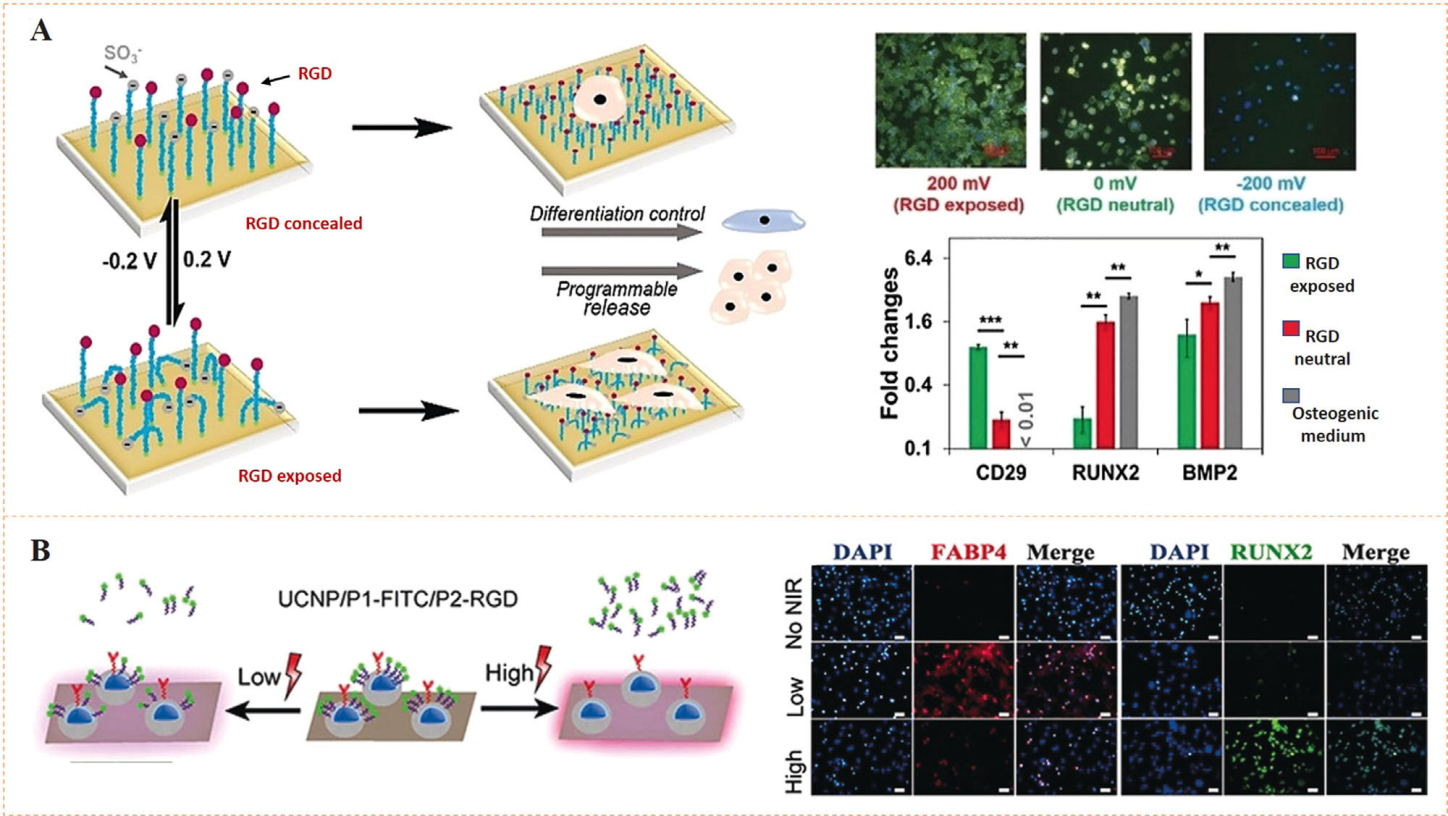
(A) The mechanism (left) of potential‐responsive surfaces for cell adhesion, release, and differentiation. Fluorescent images showed different cell adhesion behavior and the RT‐qPCR results indicated different osteogenic differentiation under different RGD exposed levels. Reproduced with permission.^[^ ^53^ ^]^ Copyright 2019, John Wiley and Sons. (B) Schematic illustration (left) of the photocleavable substrate exposed to no NIR, low‐power NIR, or high‐power NIR irradiation. Immunofluorescence imaging (right) of biomarkers for osteogenic (RUNX2, green) and adipogenic (FABP4, red) differentiation under different NIR exposures. Reproduced with permission.^[^ ^152^ ^]^ Copyright 2018, John Wiley and Sons

As previously mentioned, potential‐responsive bioligand presentation was mainly based on molecular screening, which could be precisely controlled by the strength and polarization of applied electric fields, leading to variation of the bioligand density. In view of this, Zhang et al. reported a potential‐responsive SAM for bi‐directional control of stem cell differentiation (Figure 19A).^[^ ^53^ ^]^ In addition to reversible cell adhesion modulated by applying potentials, adherent stem cells on the SAM showed distinct directions of differentiation. For example, a high accessible RGD under positive potential might lead to osteogenesis, but a buried RGD ligand induced by negative potentials was more likely to produce adipogenesis. According to the findings, potential‐controlled variations in “effective RGD” density might influence stem cell differentiation. This work thus provides another remote strategy to determine stem cell fate, which is potentially useful to direct in vivo cell differentiation and promising in tissue engineering and regenerative medicine.

Aside from magnetic and electric fields, light has also been used for switching bioligand. Considering the invasiveness and poor tissue penetrability of UV light, researchers have been inclined to use NIR light for dynamic bioligand presentation. For example, NIR photoresponsive substrate for manipulation accessibility of RGD ligand was reported, and the effect of tunable RGD‐integrin binding on multidirectional stem cell differentiation was studied (Figure 19B).^[^ ^152^ ^]^ The photoresponsive system was based on UV‐cleavable ONA and UCNP. The UCNP was modified with PEG‐RGD and ONA‐linked PEG. An NIR (980 nm) upconverted to local UV light could lead to the cleavage of ONA and PEG release. This process could gradually expose the RGD ligand for cell membrane integrin. The authors found that a low‐power NIR irradiation led to less RGD exposure, which was inclined to direct stem cells differentiation into adipocytes. Upon irradiation under NIR with the higher power, more RGD exposed and stem cells on the substrate exhibited a high level of osteogenic differentiation. This strategy may also be used for remote control of multidirectional stem cell differentiation, showing great promise for on‐demand directing of tissue regeneration in vivo.

### 3.4 Pathogenic cell isolation

Cancer metastasis has proven to be highly associated with the detachment of malignant cells (i.e., CTCs) from a solid tumor and their re‐attachment via the peripheral blood circulation system or lymphatic system.^[^ ^133^ ^]^ As a biomarker of clinical cancer, the detection and analysis of these pathogenic cells have very important clinical research significance on tumor metastasis, recurrence, or prognosis, because it is projected to be a powerful tool for real‐time treatment stage monitoring and drug treatment evaluation for tumor metastasis mechanism and drug development.^[^ ^10^, ^153^ ^]^ Cancer cell isolation requires both a particular cell‐binding process and follow an efficient release for cell collection. Therefore, it is very attractive to reversibly present cancer‐cell‐targeting ligands at the dynamic biointerfaces.

Cancer‐targeting small molecules, peptides, antibodies, and DNA aptamers have been widely used in selective cell capture and release in the field of dynamic biointerfaces.^[^ ^154^ ^]^ In addition to cancer cell selectivity, the release of captured cells represents another challenge for CTC analysis. To obtain CTCs with their original phenotypes or functions, the releasing processes are required to be with both high efficiency and friendliness. To date, external stimuli like light, pH, temperature, electric potential, chemicals, and biomolecules have been employed in dynamic biointerfaces for cell release. However, most of them feature non‐natural stimulations. The changes of CTCs phenotypes or functions probably affect the accuracy of diagnostics in the next cell analysis step. Recently, researchers have focused on the high efficiency and biocompatibility of CTC isolation. Especially, biological‐responsive surfaces capable of triggering cell release through near‐physiological stimuli or biomolecules attract wide attention.

**FIGURE 20:**
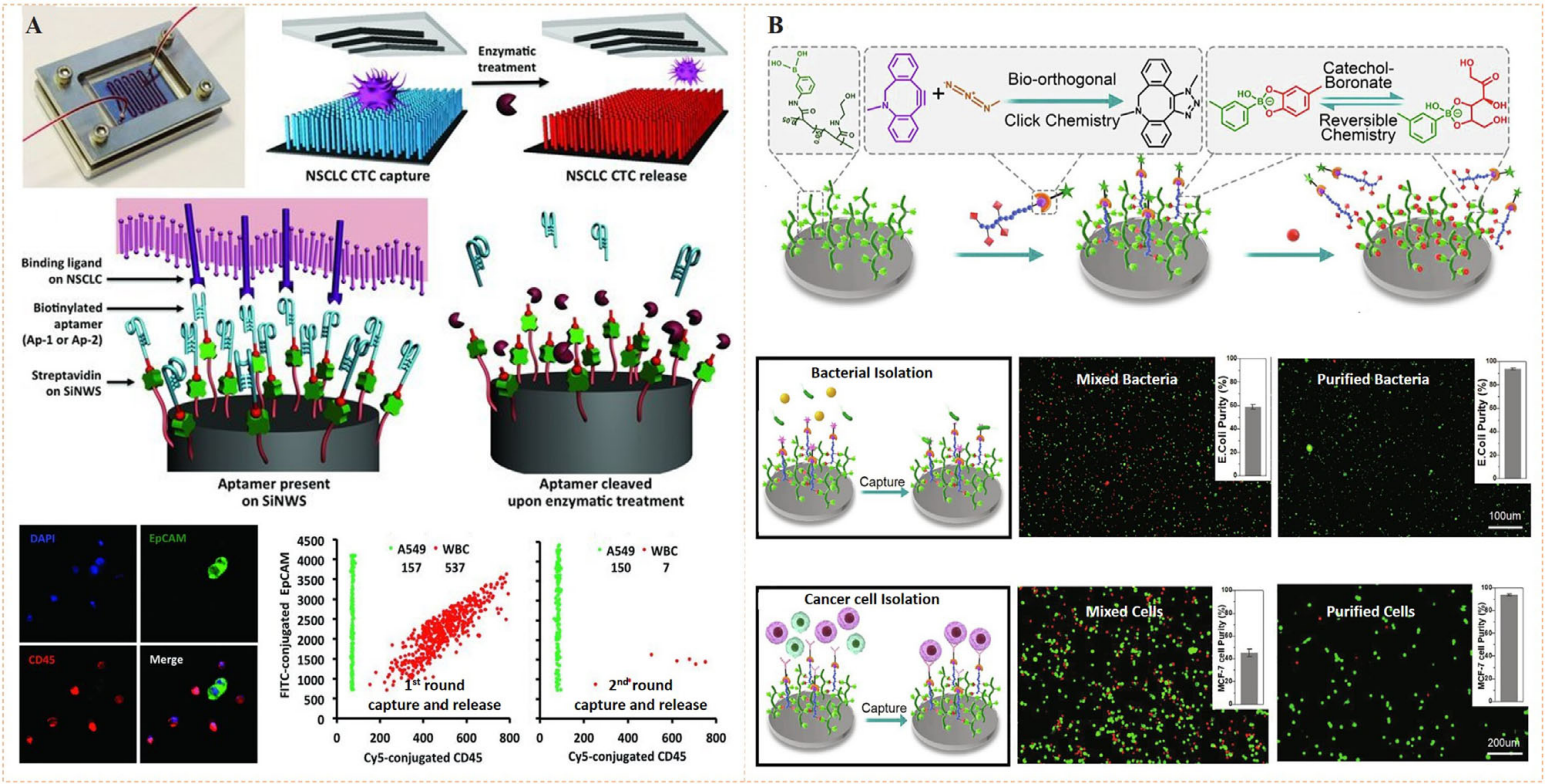
(A) The aptamer‐coated NanoVelcro Chip for capturing and releasing CTCs from blood samples. Upon treatment with the enzyme, the surface‐grafted aptamers were specifically cleaved, resulting in a release of the captured CTCs from the aptamer‐coated SiNWS. Bottom shows the capture and purification of A549 cells from blood samples. Reproduced with permission.^[^ ^21a^ ^]^ Copyright 2015, John Wiley and Sons. (B) Chemical mechanism of the surface for dynamic presentation of different bioligands. The surface showed potential as a diverse platform for both bacteria and cancer cell capture and release. Reproduced with permission.^[^ ^68^ ^]^ Copyright 2020, Elsevier B.V

Shen et al. reported a new‐generation NanoVelcro Chip for CTCs isolation from blood with high‐efficiency capture and endonuclease‐responsive release (Figure 20A).^[^ ^21a^ ^]^ The chip was based on a silicon nanowire substrate (SiNWS) modified with cancer‐targeting single‐stranded DNA aptamers. The authors demonstrated the NanoVelcro Chip enables the capture of blood CTCs without contaminating the surrounding white blood cells as little as possible. Moreover, due to the endonuclease‐triggered specific degradation of grafted DNA aptamers, the release process brought negligible damage to the viability and functions of collected CTCs. In another interesting work, Tian et al. designed a dynamic and magnetic micro‐platform with glucose responsiveness for selective cancer cell isolation. The micro‐platform was fabricated by reversibly introducing a catechol‐rich cancer‐targeting peptide on PBA‐functionalized magnetic microbead through the constitution of dynamic catechol‐PBA complexes. The magnetic micro‐platform possessed sugar‐switchable cancer‐targeting activity, owing to the glucose‐sensitivity of catechol‐PBA covalent bonds. This thus enabled magnetic capture and release of cancer cells via a natural biofeedback mechanism (i.e., the human glycemic volatility). The authors discovered that the micro‐platform could magnetically and effectively remove rare cancer cells from medium and blood samples, suggesting that it has the potential to be used as a generic tool for identifying and separating uncommon CTCs as well as diagnosing cell‐based illness. We expect further efforts on bioresponsive dynamic strategies will be continuously developed for noninvasive isolation of CTCs in the future.

Apart from CTCs, bacteria are another kind of pathogenic cells with serious threats to public health. The isolation and analysis of pathogenic bacteria are also significant for infection diagnostics, prevention, and therapies.^[^ ^155^ ^]^ For dynamic biointerfaces, the bacterial capture and release mechanism is the same as that of CTCs isolation. For instance, selective bacteria capture and on‐demand release could be realized by photoresponsive host–guest supramolecular chemistry,^[^ ^93a^ ^]^ thermo‐responsive polymer,^[^ ^156^ ^]^ reversible PBA‐*cis‐*diol covalent interaction,^[^ ^68^ ^]^ peptide assembly,^[^ ^157^ ^]^ and so on. In theory, all the methods used for CTCs isolation are suitable for all kinds of bioparticles including bacteria and even viruses. The hypothesis was conceptually proved by Tian and co‐workers (Figure 20B).^[^ ^68^ ^]^ They combined dynamic covalent chemistry with bioorthogonal click reaction, and developed a versatile platform with optional and diversified bioactivities for capturing and releasing various pathogenic bioparticles. This work provides a facile strategy for simulating the diversity of ECM dynamics and a general platform in the bioseparation diagnostics and therapeutics.

### 3.5 Single cell analysis

Cell analysis is a very useful strategy to understand and predict the factors underlying various cellular processes like health, proliferation, function, and death. The heterogeneity of cells is a big roadblock for cell‐based analysis. It is still a great challenge to analyze the heterogeneity of cancer cells through morphological and phenotypic profiles in cancer diagnosis and treatment. Fortunately, the emergence of single cell technology brings a powerful tool to solve this problem. Single cell assays can reveal the diversity of cellular behavior by analyzing transcription, translation, regulatory, and signaling events within individual cells at the molecular level. For example, it can analyze the heterogeneity of tumor cells, reveal the mechanism of tumor metastasis, study its epigenetic changes and guide the individualized treatment strategy.^[^ ^158^ ^]^

**FIGURE 21:**
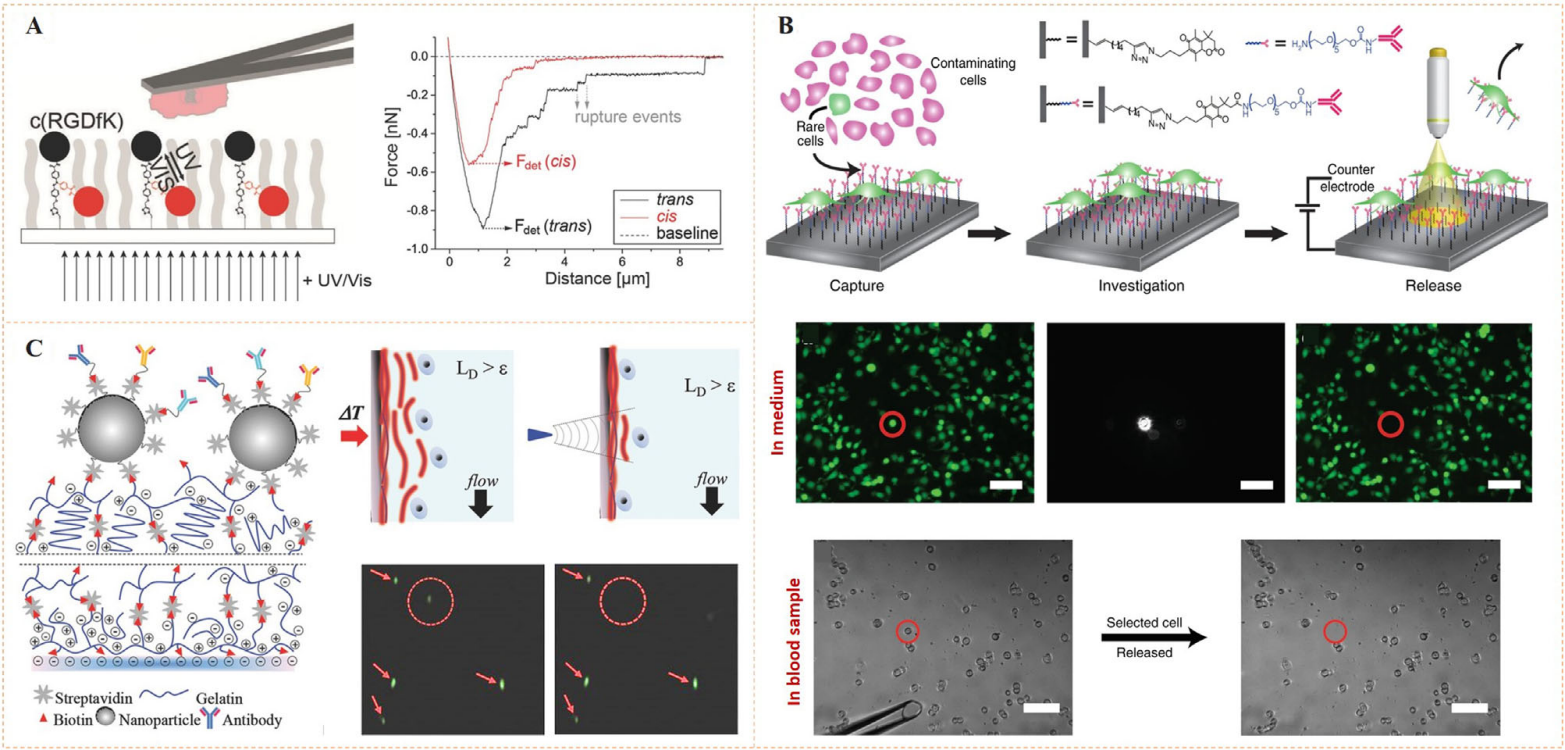
(A) Single cell force analysis (left) and the spectroscopy (right) of RGD‐mediated cell adhesion on an azobenzene‐based photoswitchable surface. Reproduced with permission.^[^ ^24c^ ^]^ Copyright 2015, John Wiley and Sons. (B) The mechanism of the photo‐electrochemical capture and release device. Rare cells (green) could be selectively captured, simultaneously analyzed, and electrochemically released. In both cell culture medium and blood sample, single target cell could be identified and released with high precision. Reproduced with permission.^[^ ^42b^ ^]^ Copyright 2018 Nature Publishing Group. (C) Schematic of the modified nanocoating with bulk release mechanism and the single cell/selective release mechanism. Fluorescence microscopy images showed the single cell release from the nanocoating. Reproduced with permission.^[^ ^159^ ^]^ Copyright 2015, John Wiley and Sons

The combination of dynamic biointerfaces with micromanipulation techniques provides the possibility to control single cell behaviors in a localized position, showing the promise in single cell analysis. For instance, integrin‐mediated cell adhesion forces could be analyzed by using single cell force spectroscopy (SCFS).^[^ ^24c^ ^]^ In a typical SCFS test, single cell adhered to a cantilever was repeatedly exposed to an azobenzene‐based photoresponsive dynamic biointerface. The cantilever deflection thus can be used to quantitatively evaluate the dynamic single cell adhesion forces (Figure 21A). This quantitative study on single cell adhesion would provide more intuitive information for regulation of cell–material interaction biomechanical research. Beyond that dynamic biointerface with photocontrolled bioligand, presentation also allows localized modulation of cell–material interactions, showing the potential to control single cell attachment and detachment. This technology allows for the separation and examination of single cells, and also the collecting of uncommon cells for expansion and future research.

Nevertheless, the above study implied that the azobenzene isomerization cannot completely release the attached cells, according to the force curves in Figure 21A. This is probably because the isomerization cannot completely shield the RGD ligands. From this point of view, a photocleavable dynamic biointerface is superior to others. Recently, Gooding's group developed a photoelectron‐responsive dynamic biointerface based on light‐activated electrochemistry for single cell isolation (Figure 21B).^[^ ^42b^ ^]^ The dynamic biointerface was based on electrochemically cleavable moiety, and the use of semiconducting electrodes in depletion allowed electrochemically activate a specific region of the surface by shining light, because only the site of illumination has sufficient charge carriers.^[^ ^160^ ^]^ This could avoid the use of electric wires to connect a specific location of the surface. The combination of light and electrochemical potential facilitated the observation of a single cell and release of them as desired. The author proposed the strategy used in this work would be very useful in drug discovery, because it could provide insight into the drug‐affected cells with both qualitative and quantitative information.

In addition to photocontrolled single cell release, Reátegui et al. reported a nanocoated microfluidic CTCs isolation device for not only temperature‐responsive release of bulk‐population cells but also the mechanosensitive release of single cell (Figure 21C).^[^ ^159^ ^]^ The nanocoating was formed by self‐assembly of biotin labeled gelatin and streptavidin with polystyrene nanoparticles on plasma‐activated PDMS (polydimethylsiloxane) surface. Gelation is themosensitive, thus allowing temperature‐triggered release of all the attached cells (37℃) in a few minutes. More interestingly, mechanical stress applied by a frequency‐controlled microtip might disintegrate isolated portions of the nanocoating as well. This localized mechano‐sensitivity thus allowed a single cell release process. The above studies jointly demonstrated that dynamic presentation of bioligands with localized controllability is applicable for the regulation of single cell behavior and especially single cell analysis. Further efforts are highly anticipated in this area as the accuracy of cell analysis is required to be increasing with the development of modern medicine.

### 3.6 Tissue engineering

Tissue engineering aims at the regeneration of neotissues from cells with the support of biomaterials and growth factors.^[^ ^161^ ^]^ Thereinto, biomaterials play a pivotal role. Ideal biomaterials are expected to work as the ECM to conduct tissue repair by eliciting desired cellular functions and modulating cell–cell/cell–material interactions.^[^ ^162^ ^]^ As ECM mimics, dynamic biointerfaces enable the manipulation of diverse cell behaviors including cell adhesion, migration, differentiation, and so on. Thus, applying the concept of dynamic ECM ligand presentation in current biomaterial design has significant implications in tissue engineering and regenerative medicine.

A typical study, reported by García and co‐workers, demonstrated that poly(ethylene glycol) diacrylate (PEGDA) hydrogel surface with photocaged control of bioligand display could modulate inflammatory responses, fibrous encapsulation, and vascularization in vivo through subcutaneous implantation of a photocaged hydrogel.^[^ ^41^ ^]^ This study suggested the possibility of photoactivated biomaterials for remote control specific cell–biomaterials interaction in vivo and improve the outcomes of biomaterials for tissue repair. Despite the great potential, the UV‐light‐dependent ligand cage in this study actually is limited in practical applications in view of the spatial resolution and tissue penetration of UV light. NIR shows enhanced tissue penetration and can reduce light scattering. However, the NIR‐responsive ligand presentation has not been employed yet for in vivo cell regulation and tissue regeneration. Further efforts on the development of NIR‐^[^ ^39^, ^152^ ^]^ or magnet‐responsive^[^ ^135^, ^137^ ^]^ or magnet‐responsive dynamic systems capable of remote modulation of in vivo cell–material interactions are urgently needed.

**FIGURE 22:**
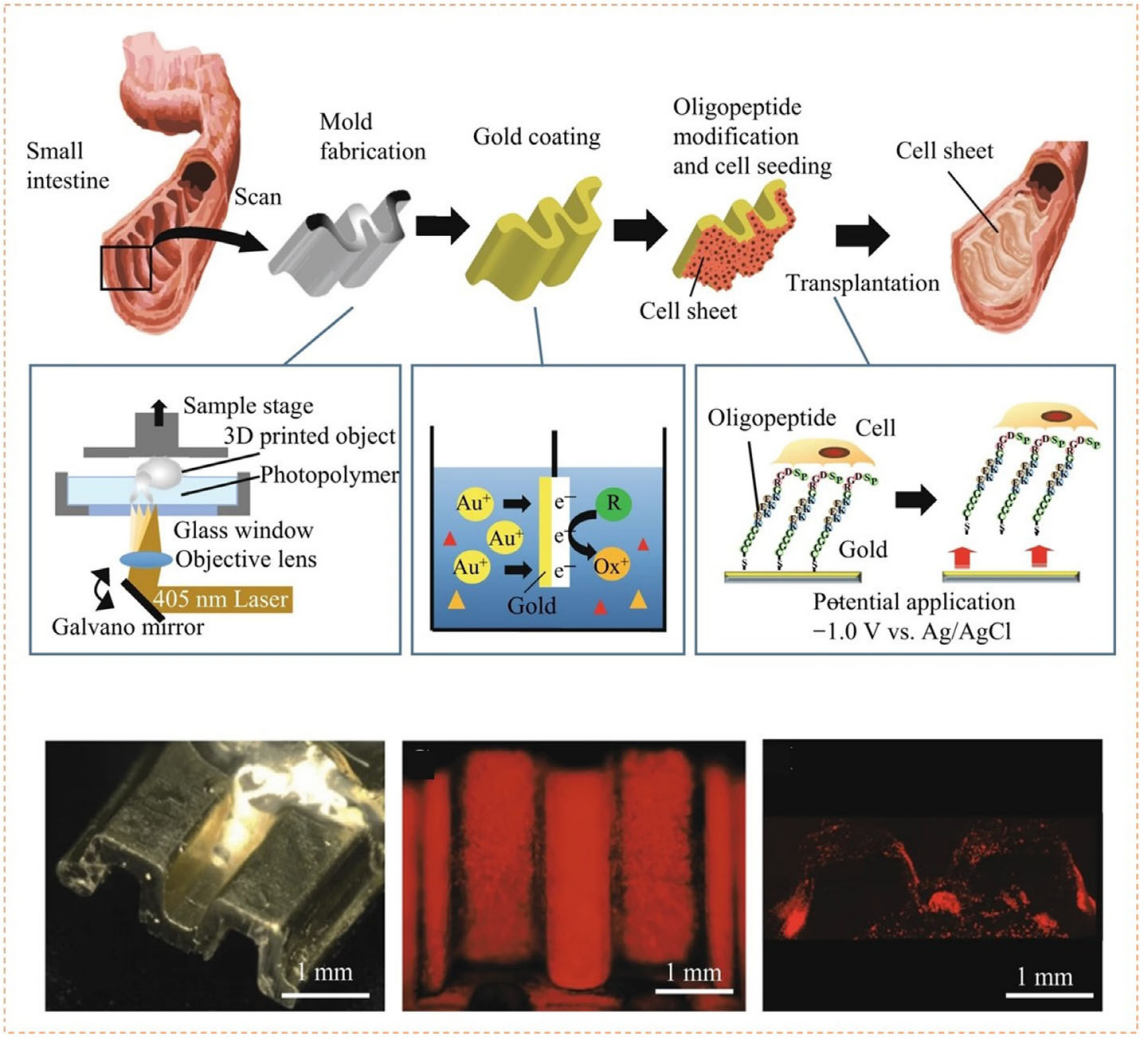
Strategies for transplanting tailor‐made cell sheets using electrochemical cell detachment. 3D mold was fabricated using microstereolithography corresponding to a surgery site. After electroless gold plating, oligopeptide coating, cell seeding, and cell confluence, cell sheet transplantation was performed using electrochemical cell detachment. Reproduced with permission.^[^ ^87c^ ^]^ Copyright 2019, Nature Publishing Group

Another potential of dynamic biointerfaces in tissue engineering and regenerative medicine is the reversible cell adhesion regulation for harvesting cell sheets.^[^ ^18^, ^87^, ^163^ ^]^ Cell sheet engineering has proven to be an emerging and efficient approach for cell transplantation.^[^ ^164^ ^]^ Typically, cell sheets can be obtained by detaching confluent cells from a thermo‐responsive material through the variation of surface wettability. Likewise, dynamic biointerfaces with reversible presentation of cell adhesive ligands can also be used to cultivate confluent cells and release them to obtain intact cell sheets. Pan et al. demonstrated for the first time an RGD‐imprinted thermoresponsive dynamic biointerface could not only control reversible cell adhesion, but also noninvasively harvest cell sheet.^[^ ^123^ ^]^ Recently, Fukuda's group further developed a method for recovery of 3D configuration‐tailored cell sheet through electrochemical dynamic surface based on Au‐S coordination (Figure 22).^[^ ^87c^ ^]^ To obtain tailor‐made cell sheets with 3D configurations, microstereolithography was used with the structural information of a specific tissue obtained from computer tomography. They fabricated an Au‐coated electrode mold with surface structure matching the folds of small intestine inner wall. In this case, surface coordination with cell adhesive bioligands would facilitate the formation of 3D cell adhesive layer. Upon applying an electric potential, a tailored cell sheet could be obtained, and the resultant cell sheet enabled a direct transplantation into the diseased site of small intestine. This study proposed a realistic technique for 3D cell sheet manufacture and transplanting for curing complex tissue injuries. Although further exploration is necessary, the study still demonstrated the great potential of ECM‐mimicking dynamic biointerfaces in future tissue engineering.

## 4 CONCLUSION AND OUTLOOK

As a typical characteristic of biochemical cues in the ECM, dynamic bioligand presentation is associated with various physiological and pathological processes. As the ECM substitutions, ideal biomaterials for biomedical uses should possess the ability to recapitulate the dynamics of ECM ligands. In this context, dynamic biointerfaces were developed, as a proof‐of‐concept model, to mimic the dynamic ECM bioligand presentation on biomaterial interfaces. Reversible molecular strategies have been proven to be the efficient means to dynamically conjugate bioactive molecules on the biomaterial surfaces and to fabricate diverse dynamic biointerfaces. As discussed above, currently available dynamic strategies can be divided into covalent and noncovalent methods (Table 1). The covalent methods relied on stimuli‐triggered breakage or structural change of the dynamically covalent‐linked bioligands, while noncovalent methods for bioligand conjugations have the congenitally reversible. Almost all these developed dynamic biointerfaces could respond to external stimuli (e.g., light, temperature, pH, potential, chemicals, peptides, DNAs, and enzymes), leading to controlled bioligand presentation. To date, dynamic biointerfaces have been demonstrated to reversibly cell adhesion, control macrophage polarization, and direct stem cell differentiation. Due to the great potential to modulate cell–biomaterial interactions and manipulate cell behaviors, dynamic biointerfaces have shown the values in drug discovery, disease diagnosis, and regenerative medicine. However, a critical problem demanding prompt solution is the biocompatibility or bioapplicability of these dynamic biointerfaces. External stimuli used in these systems are mostly unphysiological and potentially invasive to living cells; only a small part of them is involved in natural biological processes. It is also worth mentioning that the control of cell adhesion or release has been extensively reported, while other cell behaviors, like migration, differentiation, autophagy, and apoptosis that are also significant in physiological and pathological processes, have been less explored. Therefore, engineering of smart biomaterial interfaces with dynamic bioligand presentation, near‐physiologically responsiveness, and noninvasive control of diverse cell behaviors still is a great challenge.

**TABLE 1:** Reversible molecular strategies for dynamic bioligand presentation at biomaterial interfaces

Category	Reversible interactions		External stimulus	Cell regulation	References
**Noncovalent strategies**	Host–guest chemical recognition	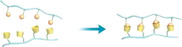	Chemical exchange, UV, electric potential	Cell/bacterial adhesion, migration, polarization, CTC isolation	^[^ ^14^, ^16^, ^24^, ^90^, ^92^, ^93^, ^94^, ^97^, ^136^, ^145^ ^]^
	DNA aptamer base pairing	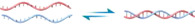	DNA chain, ATP molecule, NIR, pH value	Cell adhesion, differentiation, proliferation, CTC isolation	^[^ ^98^, ^99^, ^101^, ^102^, ^103^, ^104^ ^]^
	Tunable intermolecular interaction	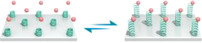	Temperature, electric potential	Cell adhesion, cell sheet recovery	^[^ ^114^, ^115^, ^116^, ^120^, ^123^, ^159^ ^]^
	Molecularly imprinted biorecognition	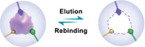	Temperature, peptides, sugar molecule	Cell adhesion, cell sheet recovery, CTC isolation	^[^ ^71^, ^123^, ^125^ ^]^
	Library‐screened biorecognition	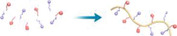	Biomolecule	Cell differentiation	^[^ ^131b^ ^]^
**Dynamic covalent strategies**	Azobenzene photoisomerization	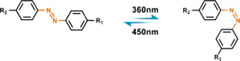	UV	Cell adhesion, bacterial adhesion	^[^ ^17^, ^22^, ^28^, ^29^ ^]^
	Nitrophenyl photocleavage	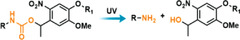	UV, NIR	Bacterial adhesion, cell adhesion, proliferation, migration	^[^ ^34^, ^35^, ^36^, ^37^, ^38^, ^39^, ^41^, ^152^ ^]^
	Quinone electrochemical redox reaction	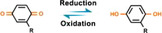	(Photo)electric potential	Cell adhesion, cell differentiation, cell pattern	^[^ ^42^, ^44^, ^45^, ^46^ ^]^
	Potential‐controlled molecular shielding	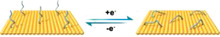	Electric potential	Cell adhesion, bacterial adhesion, cell differentiation	^[^ ^49^, ^50^, ^51^, ^53^ ^]^
	Dichalcogenide molecular exchange	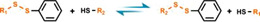	GSH	Cell adhesion, CTC isolation	^[^ ^55^, ^56^ ^]^
	Phenylboronate ester exchange	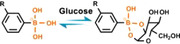	Sugars, pH value	Cell adhesion, CTC isolation, bacterial adhesion	^[^ ^65^, ^66^, ^68^ ^]^
	Imine exchange and acidolysis	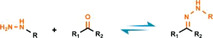	pH value	Cell adhesion, CTC isolation, cell spheroid formation, gene transfection	^[^ ^72^, ^73^ ^]^
	Enzymolysis	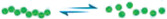	Enzyme	Cell adhesion, differentiation, CTC isolation	^[^ ^21^, ^74^, ^75^, ^76^ ^]^
	Metal coordination	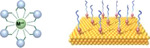	EDTA, metal ion, electric potential	Cell adhesion, cell sheet and tube formation, CTC isolation	^[^ ^80^, ^81^, ^82^, ^83^, ^84^, ^85^, ^86^, ^87^, ^135^, ^137^ ^]^

Despite these apparent difficulties, the authors are still optimistic since tremendous progress has been achieved in the last decade. Various novel strategies, such as the use of natural molecular recognition,^[^ ^50^, ^165^ ^]^ the combination of covalent and noncovalent means,^[^ ^166^ ^]^ and even protein engineering, have emerged to specifically address current issues and bring new opportunities in biomedicine. Moreover, current efforts for dynamic bioligand display have undergone an evolution from 2D surface to 3D network.^[^ ^151a^ ^]^ The integration of dynamic bioligand presentation into the 3D network of hydrogels may confer properties and functions to these hydrogels that more closely resemble the natural ECM. Based on the above successful examples, we believe that dynamic bioligand presentation on the biomaterial interfaces is approaching to be perfectly functioning replicates of the natural ECM, and these ECM mimics will play an increasingly significant role in biomedicine.

## CONFLICT OF INTEREST

The authors declare no conflict of interest.
